# Harnessing synthetic microbial communities for desert vegetation restoration

**DOI:** 10.1016/j.synbio.2026.08.013

**Published:** 2026-09-19

**Authors:** Panpan Zhang, Haibing Wang, Xiaopeng Jia, Xiaopei Zhang

**Affiliations:** a State Key Laboratory of Loess Science, Institute of Earth Environment, Chinese Academy of Sciences, Xi'an, 710061, China; b College of Desert Control Science and Engineering, Inner Mongolia Agricultural University, Hohhot, Inner Mongolia, 010018, China; c State Key Laboratory of Water Engineering Ecology and Environment in Arid Area, Inner Mongolia Agricultural University, Hohhot, Inner Mongolia, 010018, China; d State Key Laboratory of Ecological Safety and Sustainable Development in Arid Lands, Northwest Institute of Eco-Environment and Resources, Chinese Academy of Sciences, Lanzhou, 730000, China; e University of Chinese Academy of Sciences, Beijing, 100049, China

**Keywords:** Desert vegetation restoration, Soil microbes, Plant-microbial-soil interactions, Water use efficiency, Synthetic microbial communities

## Abstract

The core bottleneck for desert vegetation restoration is extreme water stress during early seedling establishment, with hydraulic failure as the primary lethal mechanism. Traditional engineering measures (e.g., irrigation) and single-strain inoculation cannot adequately address these challenges due to high cost, weak sustainability, or limited functionality. Synthetic microbial communities (SynComs) offer a new approach to synergistically regulate plant water use and soil microenvironment by integrating functionally complementary taxa. This review discusses the soil water characteristics of deserts and the bottlenecks in seedling water use, indigenous microbial resources and their adaptive mechanisms, principles and design strategies for constructing SynComs, as well as the mechanisms by which SynComs improve seedling water use efficiency and survival. We also analyze key challenges and future research directions. Although SynComs have been extensively studied for enhancing plant stress resistance, their application in desert vegetation restoration is still in its infancy. Future research needs to clarify how to design SynComs to ensure their ecological compatibility, persistence, and functionality in the highly variable and extreme environments of desertified landscapes. This review provides a theoretical basis for advancing SynComs application in arid land ecological restoration.

## 1 Introduction

Desertification is a major global ecological crisis, with desert ecosystems covering more than one-third of the Earth's land surface and directly threatening the survival and development of approximately one-fifth of the global population [1,2]. As the most structurally simplified and least stable type of terrestrial ecosystem, deserts are chronically subjected to the effects of multiple extreme abiotic stresses, including unpredictable pulsed precipitation, intense solar radiation, nutrient-poor and generally salinized soils, as well as drastic diurnal and seasonal temperature fluctuations [3]. Despite these harsh living conditions, deserts harbor highly specialized plant, animal, and microbial taxa, constituting a unique and rich treasure trove of biodiversity [4]. Notably, deserts are not only important carriers of biodiversity but also critical parts in the global carbon (C) cycle. The complex interactions among vegetation, soil, and climatic factors in desert environments create unique conditions for C capture and long-term sequestration [5]. Recent research has confirmed that stimulating plant growth plays a secondary role in regulating C storage, whereas reducing surface soil C loss by inhibiting wind erosion during dryland restoration is the primary reason for high soil C storage. This process contributes a soil C accumulation rate (7.87 Tg C yr^−1^) equivalent to 38.8% of afforestation and 56.2% of forest protection in China [6]. This indicates that deep-rooted vegetation in arid regions not only sequesters substantial C in deep soil layers, but more importantly, promotes soil C storage by reducing soil erosion, thereby making significant contributions to climate change mitigation in arid systems.

However, under the dual pressures of human activities and climate change, the continuous expansion of desertification on a global scale is constantly eroding this invaluable ecological and C sink resource [7]. The severe wind erosion processes in desertified lands not only directly lead to substantial loss of surface soil C, releasing stored C in the soil, but also reduce the quality and availability of water resources, destroy the habitat of native specialized species, and trigger a sharp decline in biodiversity [7,8]. Simultaneously, the spread of mobile sand dunes increases the risk of regional ecological disasters, threatens surrounding farmlands, grasslands, and residential safety, reduces crop yields, and consequently exacerbates poverty and economic disparities [9]. Thus, the management and ecological restoration of desertified lands are not only an urgent necessity for maintaining regional ecological security and ensuring human survival and development, but also an important pathway for enhancing global C sink and mitigating climate warming. This holds irreplaceable significance for promoting the sustainable development of ecosystems.

Among all desertification control methods, biological measures are the most dynamic component, constructing vegetation barriers by planting psammophytes to curb desertification at its source. This vegetation plays a fundamental role in maintaining the integrity of soil systems in arid regions by stabilizing sand dunes, reducing wind erosion, retaining soil moisture, improving soil quality, regulating local microclimates, providing habitats for organisms, and delivering various socioeconomic benefits to local communities [2,10]. Although vegetation restoration has significant benefits, in desert environments characterized by water scarcity, nutrient deficiency, poor soil structure, and strong evaporation, seedlings often die from hydraulic failure, which hinders the restoration process [11,12]. Therefore, although irrigation is widely used to ensure seedling establishment in sand-fixation afforestation, it is constrained by the desert environment and the underdeveloped root systems of seedlings. If irrigation management is inappropriate, such as insufficient water supply, discontinuous timing, or large fluctuations in water potential, it fails to match the water-demand patterns of psammophytic plants. This keeps plant roots in a prolonged state of starvation or drought, leading to hydraulic failure and reduced vegetation survival. Conversely, excessive irrigation (e.g., flood irrigation) can cause a continuous decline in the groundwater table and soil salinization [13]. Salt-alkali stress not only reduces soil water potential, inducing osmotic stress and impairing root water uptake, but also damages plant physiological functions through Na^+^ and Cl^−^ toxicity, thereby preventing normal water and nutrient absorption and accelerating vegetation decline [14]. More critically, irrigation provides only a temporary water supplement and cannot alleviate the long-term nutrient deficiency in sandy soils. The persistent water-nutrient coupling constraint continuously disrupts the hydraulic steady state of seedlings, leading to post-establishment dieback and failure, making restoration unsustainable. These factors underscore the urgent need to develop auxiliary biological strategies that can reduce irrigation dependence and improve water-use efficiency (WUE). Studies have shown that soil microbial communities can significantly improve soil structure and fertility through multiple mechanisms, including biological soil crusts (BSCs) formation, mycelial network construction, extracellular polymeric substance (EPS) secretion, nutrient mobilization, and microbially induced carbonate precipitation (MICP) [15], thereby providing a survival foundation for vegetation establishment in desertified areas. As the second genome of plants, microorganisms promote root growth by secreting phytohormones and metabolites [16], activate antioxidant pathways to alleviate oxidative damage, and maintain normal physiological metabolism and water uptake under water-stress conditions [17]. In addition, arbuscular mycorrhizal fungi (AMF) greatly expand the root absorption zone through their extraradical hyphae, enabling plants to acquire water and nutrients far beyond the root zone, which is crucial for plant survival in poor desert soils [[18], [19], [20]].

Indigenous desert microorganisms, shaped by long-term selection under extreme conditions (persistent drought, salinization, freeze-thaw cycles, and pulsed precipitation), have evolved highly specialized adaptive strategies. Despite their low biomass, they possess strong drought tolerance, an efficient dormancy-reactivation resilience mechanism, and remarkable functional influence [2,4,21]. Studies have confirmed that desert microbial communities can rapidly respond to fluctuations in water availability and temperature [22], and maintain key ecological processes such as C and nitrogen (N) cycling even under extreme water deficit and temperature stress [[23], [24], [25]]. Moreover, sustained drought enriches drought-tolerant, oligotrophic, and spore-forming microbial taxa while reducing the abundance of copiotrophic microorganisms, thereby forming a microbial community with stress adaptability [2].

Notably, the frequent occurrence of extreme events such as drought has deeply embedded legacy effects into microbial systems [26]. Microbial communities conditioned by drought exhibit reduced sensitivity to subsequent stress and can rapidly reorganize functional groups to enhance plant stress resistance [27]. For example, multiple drought cycles enhance the drought tolerance of *Leymus chinensis* by regulating the microbial community [28]; continuous drought reduced the diversity of rhizosphere bacteria but leads to specialization, with an increase in the abundance of biofilm-forming bacteria, which in turn enhance plant drought resistance by improving soil structure and water retention [29]; the microbiome shaped by drought legacy effects can also modulate host transpiration and gene expression, improving plant WUE and drought resistance, thereby mitigating the negative physiological impacts of acute drought on plants [30]. Therefore, harnessing these microbial resources with adaptive memory represents an important approach to improving the efficiency and sustainability of desert vegetation restoration.

Studies have shown that inoculation with indigenous desert microorganisms (bacteria or AMF) can effectively promote vegetation growth in arid regions or alleviate the negative impacts of drought on soil functions [20,31,32]). For example, under desert climatic conditions, inoculation with *Bacillus pumilus* ES4, a cactus root endophyte, enhanced mesquite seedling root biomass and rhizobium populations in the same soil [33]. Rhizobacteria from the Atacama Desert plant *Cistanthe longiscapa* effectively improved the growth performance of tomato seed germination and seedling growth under water deficit stress [34]. Similarly, AMF inoculation has shown remarkable growth promoting effects: it increased the root:shoot ratio of *Artemisia sphaerocephala* and reduced salicylic acid (SA) and abscisic acid (ABA) levels, thereby benefiting seedling establishment in desert [35]; AMF strains isolated from desert plants of the Arabian Peninsula promoted the growth of *Phoenix dactylifera* L. and *Prosopis cineraria* L. (Druce) seedlings [36]; in another study, AMF inoculation significantly increased biomass accumulation, promoted root growth, and enhanced soil enzyme activity of *Elaeagnus angustifolia* L. seedlings in saline soils [37]. Collectively, these studies indicate that beneficial microbial inoculation holds considerable potential for enhancing drought tolerance and promoting vegetation restoration in desert ecosystems.

Although microbial inoculation has achieved some success in helping desert plants growth, a single strain is constrained by its narrow ecological niche and single metabolic pathway. Regardless of its capacity for phosphorus (P) solubilization, N fixation, or EPS production, its intervention is essentially a single-target functional approach. In contrast, synthetic microbial communities (SynComs) (Table 1) represent simplified modular tools with relatively complex structures, featuring interspecies metabolic interactions, functional division of labor, and ecological cooperation, and their overall efficacy in complex environments is generally significantly higher than that of individual strains [38,39]. Through the rational assembly of functional strains occupying different ecological niches such as EPS-producing strains to enhance soil water retention, N-fixing and P-solubilizing strains to activate nutrients, PGPR to regulate stomatal behavior and root development, and AMF to expand the water absorption interface, it is possible to simultaneously alleviate the water-nutrient coupling limitation in vegetation [2]. Furthermore, in contrast to individual strains, SynComs exhibit key advantages such as functional redundancy, environmental adaptability, and robustness [40]; relative to natural microbial communities, SynComs are characterized by lower complexity, greater controllability, and higher reproducibility [41]. Currently, direct evidence for SynComs-mediated alleviation of seedling water-nutrient coupling limitation in desert ecosystems remains limited. Encouragingly, however, their feasibility in enhancing drought and salt tolerance has been validated in a variety of crop, including wheat, maize, rice, tomato, watermelon, and *Astragalus* [17,[42], [43], [44], [45], [46], [47]]. Furthermore, studies on stress-resistant microorganisms screened from the roots or rhizosphere of desert plants have achieved promising results in improving crop drought and salt resistance [[48], [49], [50]]. Therefore, we believe that constructing targeted SynComs using desert stress-tolerant microorganisms holds promise for synergistically promoting plant survival and soil improvement. Meanwhile, the well-established theories, design principles, construction methods, and validation approaches accumulated in the fields of agriculture and soil remediation can provide robust support for the construction of desert SynComs, thereby effectively advancing the process of vegetation restoration.

**Table 1:** The terms and definitions provided in this study.

Term	Definition
Hydraulic failure	The cascading process in which water transport system collapses in plants due to water deficit, including root cell dehydration, xylem embolism, and whole-plant desiccation death.
Arbuscular mycorrhizal fungi (AMF)	Plant root symbiotic fungi enhance water and nutrient uptake and promote soil aggregation through hyphal networks and glomalin production, while regulating the rhizosphere microbial community to synergistically promote vegetation restoration and improve ecosystem stability.
Ectomycorrhizal fungi (EMF)	EMF form mutualistic symbioses with plant fine roots, significantly enhancing tree adaptation to drought environments by optimizing root water uptake and physiological responses.
Biological soil crusts (BSCs)	Composed of cryptogamic organisms (e.g., bacteria, cyanobacteria, fungi, lichens, and mosses), they serve as the skin of arid ecosystems, playing an irreplaceable role in maintaining soil surface stability, regulating climate, and promoting ecological restoration.
Biofilm formation	A process in which microorganisms attach to biological or abiotic surfaces (e.g., soil particles, plant root surfaces), secrete EPS, and encase themselves to form aggregates.
Synthetic microbial communities (SynComs)	An artificial ecosystem constructed from two or more microbial strains with well-defined taxonomic and functional characteristics, designed to achieve specific functions such as water retention, nutrient cycling, or alleviation of biotic and abiotic stress.
Cross-kingdom synthetic microbial communities (Cross-kingdom SynComs)	A functional microbial community rationally designed and constructed from microorganisms belonging to different biological kingdoms (e.g., bacteria, fungi, archaea) to achieve specific desired functions.
Genome-scale metabolic models (GSMMs)	Mathematical models that integrate genomic, proteomic, and metabolomic data to predict metabolic fluxes, nutrient exchange, and interspecies interactions within microbial communities.
Single-cell sorting	A process that isolates individual cells from complex microbial communities using techniques such as fluorescence-activated cell sorting and microfluidics.
Microfluidics cultivation	A method whose core principle is to construct nanoliter-scale culture chambers on an integrated chip to achieve rapid single-cell sorting. It allows precise control of culture conditions such as temperature, nutrients, and pH.
Membrane diffusion cultivation	Using permeable membranes that enable nutrients and metabolites to diffuse into the cultivation medium, while microbial cells are retained within the membrane to form pure cultures or colonies, this method closely simulates the in-situ growth environment.

To address this gap, this review is structured from four perspectives: (1) elucidating the hydrological characteristics of deserts and the water use bottlenecks faced by seedling; (2) analyzing indigenous desert microbial resources and their stress-resistance mechanisms; (3) proposing construction principles and design strategies for SynComs tailored to desert vegetation restoration; and (4) focusing on how SynComs enhance WUE and drought survival during early seedling establishment. Finally, (5) we discuss the challenges and future research directions for the application of SynComs in desert vegetation restoration (Fig. 1). In summary, most research indicates that arid regions will continue to expand, highlighting the necessity of exploring flexible strategies (e.g., SynComs) for desert vegetation recovery. The successful establishment of such resilient plants is key to tackling urgent global issues, notably climate change mitigation and land degradation. This review aims to provide theoretical support for the containment of desertification in the context of global climate change.

**Fig. 1:**
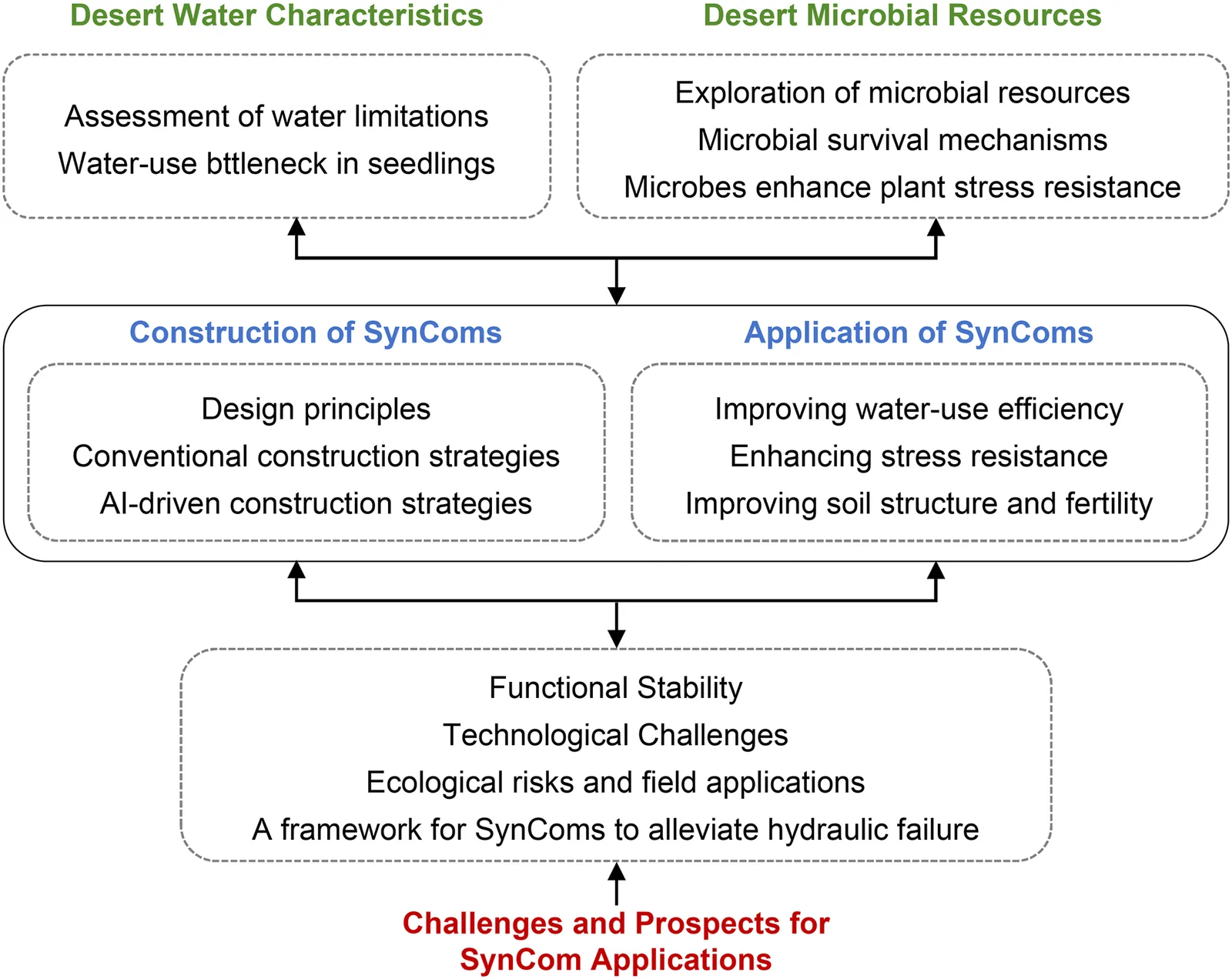
Schematic overview of the review's logical structure.

## 2 Desert water characteristics and plant water use bottleneck

Increasing temperatures and altered precipitation regimes are aggravating water scarcity in drylands through enhanced evaporative demand and diminished water supply, thereby imposing a persistent challenge for the establishment of plant seedlings in the future [51]. This section aims to elucidate the water characteristics of desert environments and their constrains on early seedling establishment (Fig. 2), providing a basis for subsequent analysis of how SynComs can be targeted to enhance plants WUE and survival.

**Fig. 2:**
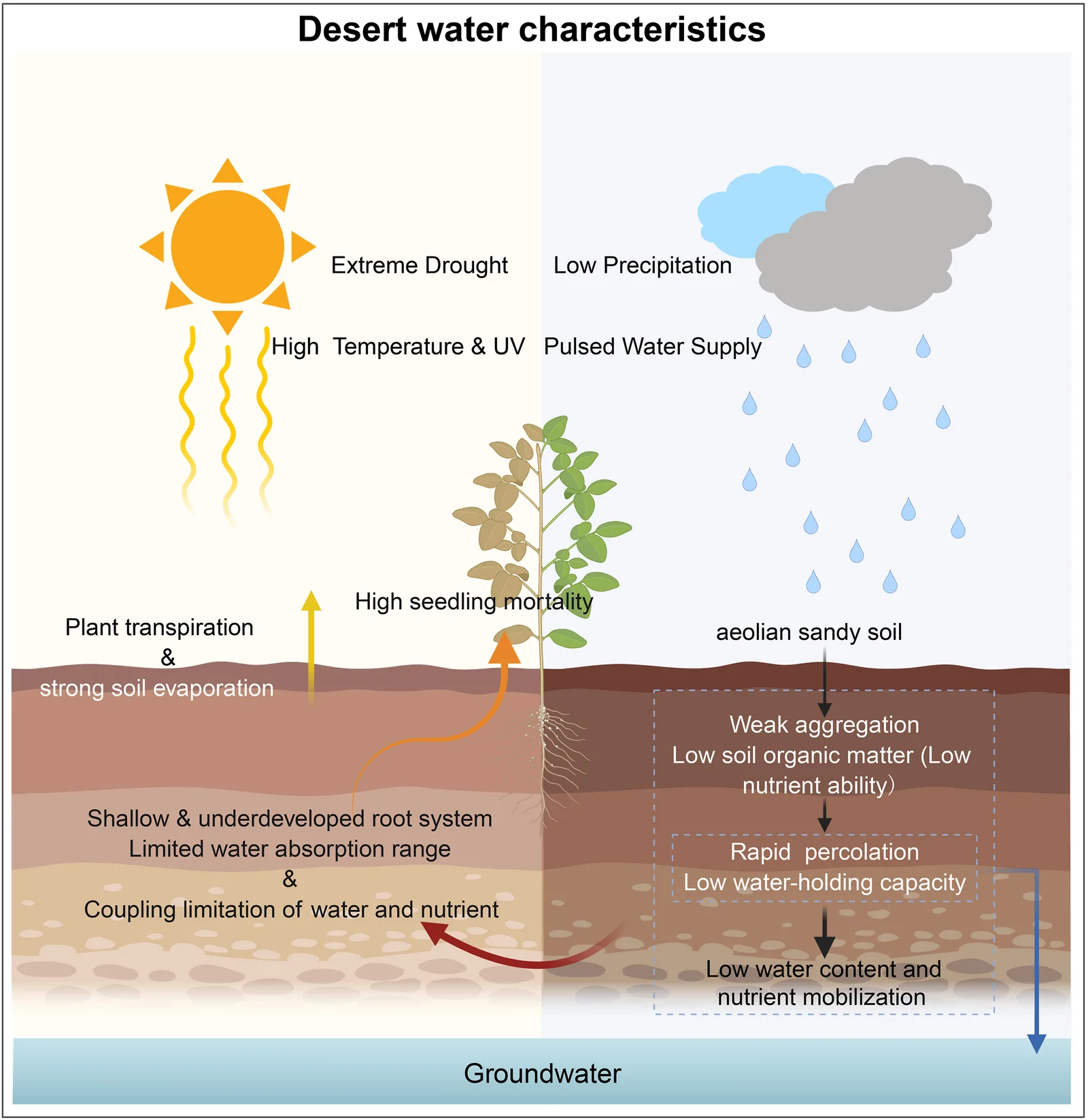
Desert water characteristics and their primary constraints on plant establishment. Pulsed precipitation, intense evapotranspiration, and deep percolation collectively shape an extreme water environment, subjecting seedlings to the dual stresses of water and nutrient limitation.

### 2.1 Desert water characteristics

Desert water is not absolutely absent, but presents the characteristics of pulse supply and rapid loss. Precipitation events in arid regions are generally characterized by discontinuity, high variability, low rainfall and short duration, and substantial interannual or seasonal fluctuations [52,53]. Consequently, precipitation in arid regions is regarded as a pulsed resource input, which affects a series of ecological responses of soil moisture, nutrient and C cycles [54]. Desert ecosystems are highly dependent on limited water resources provided by both precipitation and non-rainfall (e.g., dew) events [55]. However, due to extreme aridity and high temperatures, surface soil moisture rapidly lost through evapotranspiration and deep percolation following precipitation [56], making it difficult for seedlings with underdeveloped root systems to effectively acquire water. As sandy soils dry, the rhizosphere soil water potential drops below the root cell water potential, causing water to flow outward from root cells into the soil, leading to rapid root desiccation and death—which represents the most direct water stress faced by seedlings during the early establishment phase. In this context, dew represents a significant fraction of non-precipitation water inputs. Despite its limited single-event volume, it functions as a persistent and reliable water source, which is essential for sustaining ecological equilibrium in arid and semi-arid areas [57]. KIDRON et al. [58] observed in the Negev Desert under bare ground conditions that annual total dew accounted for approximately 36% of the annual precipitation. Chen et al. [59] demonstrated that dew under shrub canopies decreased by 29-33% compared to bare sand. Similarly, Pan et al. [60] found that dew formation in open spaces was four times higher than that under shrub canopies. These findings indicate that during the initial establishment phase, when seedlings have small canopies and the ground surface is largely bare, conditions are actually favorable for condensation formation and accumulation. Although some studies have reported higher dew under *Artemisia ordosica* shrub cover compared to bare sand, with an annual condensation amount of 47.1 mm accounting for 19.6% of annual precipitation [61], a discrepancy that may arise from differences in regional climatic conditions and vegetation types, regardless of land surface type, dew remain an important water source for sustaining plant survival during dry periods. For seedlings with shallow roots that cannot access deep groundwater, dew, which occurs frequently on bare sand surfaces, provides a critical water source—either taken up directly by leaves or absorbed by roots after infiltrating the surface soil.

### 2.2 The coupling effect of water and nutrient limitations

The water bottleneck of vegetation establishment does not persist throughout the entire growth period but is highly concentrated during the critical window period of seedling establishment and root development. In the early establishment phase, seedling roots are confined to the surface soil layer and are unable to access deeper stored water, rendering them extremely sensitive to short-term absence of precipitation. As the plant grows, roots gradually extend into deeper layers, enhancing water acquisition capacity and reducing dependence on surface soil moisture [51]. However, the constraints on seedling in deserts often arise not from water deficiency alone, but from the coupled stress of water and nutrient limitations [55]. In extremely arid environments, soil geochemical cycling proceeds slowly, severely limiting nutrient availability and mobility. Nutrient deficiency not only directly restricts vegetation growth but also impairs plant water acquisition and transport by suppressing root development. In addition, inadequate nutrient supply limits photosynthesis, thereby reducing the amount of C fixed per unit of water and consequently lowering WUE: N deficiency reduces chlorophyll content and photosynthetic efficiency [62], whereas P limitation exerts an even stronger inhibitory effect on photosynthesis—approximately 1.5 times greater than that of N limitation [63]. Studies have demonstrated the relationship between nutrient status and plant water use. For instance, Nutrient limitation, intensified the reduction in photosynthesis and WUE of the semi-arid shrub *Helianthemum squamatum*, decreasing both plant biomass and drought survival capacity [64]. During drought, reduced nutrient supply suppresses C assimilation; consequent C limitation further impedes N uptake and assimilation, while also reducing sugar availability for xylem embolism repair—and embolism, in turn, disrupts xylem-phloem water cycling, aggravating hydraulic failure or C starvation [65]. By contrast, efficient P acquisition enables *Miscanthus sinensis* to maintain higher WUE and enhance N fixation, conferring a competitive advantage under combined stresses of P scarcity, N limitation, and seasonal drought [66]. Furthermore, appropriate supplementary fertilization during early tree growth can significantly improve drought resistance and survival [67]. Collectively, these findings underscore that integrating nutrient activation with water management to break the water-nutrient coupling constraint is essential for improving seedling survival.

### 2.3 Restriction of desert soil physical properties on water retention

The physical structure of aeolian sandy soil further amplifies the water stress experienced by seedlings, constituting a structural water deficit barrier to desert vegetation establishment. Firstly, aeolian sandy soil is predominantly composed of sand particles (content can exceed 80%), with extremely low silt and clay content. This results in a loose soil texture, poor structural stability, and weak erosion resistance, making it difficult to form stable aggregate structures. Secondly, aeolian sandy soil is characterized by high bulk density and large inter-particle pores, leading to rapid water infiltration, high evaporation rates, and exceptionally poor water retention capacity—following precipitation, surface soil moisture rapidly infiltrates or evaporates, failing to effectively persist within the root zone [68]. More critically, desert soils are extremely deficient in soil organic matter (SOM), which is precisely a core element driving soil water retention capacity: SOM promotes aggregate formation and improves soil structure; furthermore, SOM itself is highly hydrophilic, and higher SOM content confers greater water retention capacity to the soil [69,70] Consequently, the sandy texture, coarse porosity and SOM deficiency of aeolian sandy soils collectively contribute to their extremely low water-holding capacity, causing rhizosphere moisture to rapidly deplete following precipitation events.

In summary, the water bottleneck for desert seedlings establishment resulted from the combined effects of pulsed water supply, coupled water-nutrient stress, and structural water retention deficiency. This indicates that vegetation establishment cannot rely solely on short-term water supplementation measures such as irrigation, but necessitates a synergistic approach addressing three levels: enhancing the soil's inherent water retention capacity, improving seedling WUE and simultaneously alleviating nutrient limitations. This provides a clear practical demand and application scenario for the introduction of SynComs.

## 3 Desert microbial resources and stress resistance mechanisms

In hyper-arid deserts, plants provide a refuge for these microbes [71]. Similarly, the highly specialized microbial communities inhabiting their rhizosphere constitute the core natural functional units for constructing SynComs. This section elucidates the desert microbial resources and their stress resistance mechanisms as well as ecological effects (Fig. 3).

**Fig. 3:**
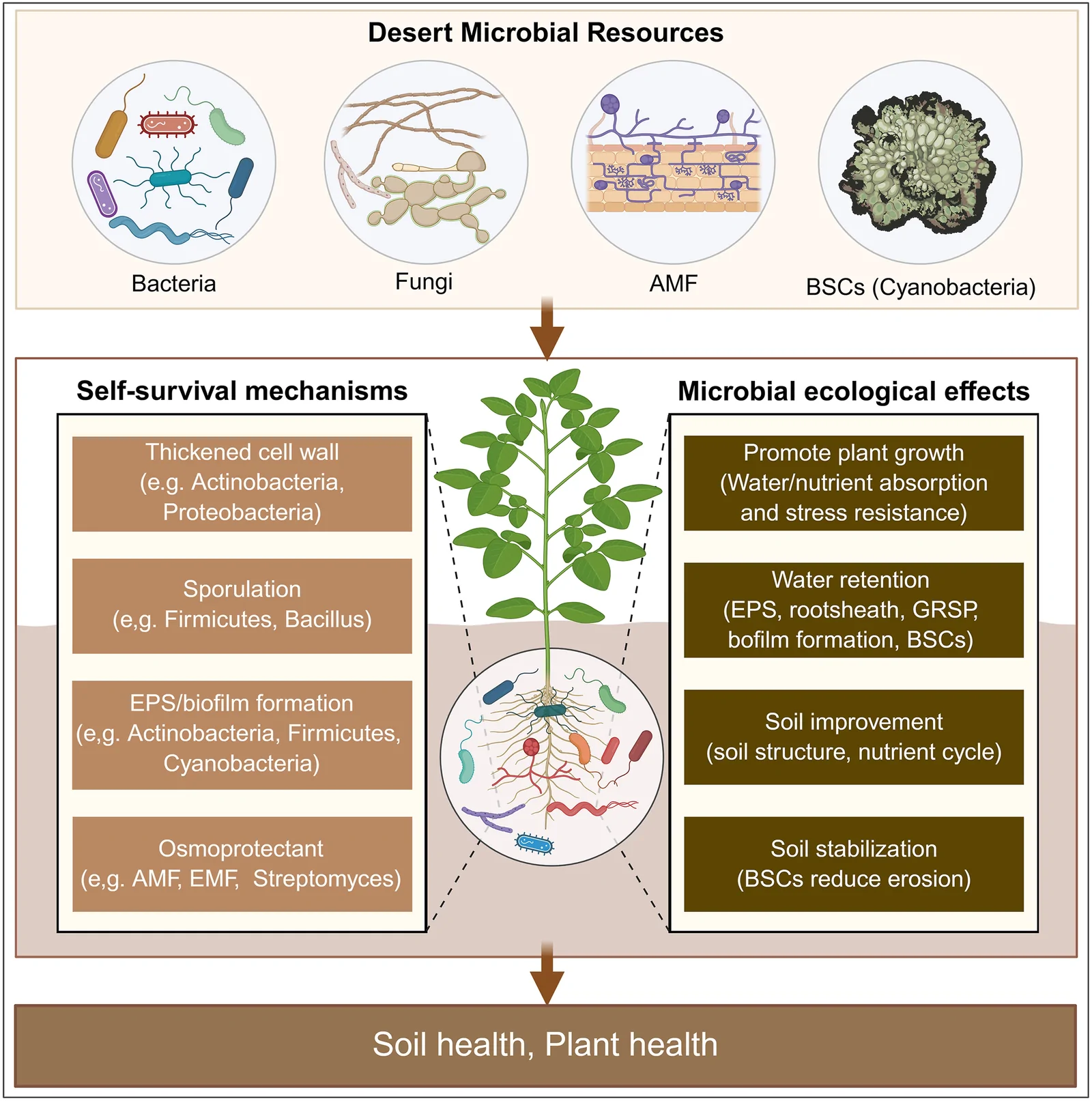
Desert microbial resources and their ecological effects. This diagram illustrates indigenous desert microbial taxa and their stress tolerance strategies (including thickened cell walls, sporulation ability, EPS secretion, and osmoprotectant accumulation), as well as their beneficial effects on soil water retention, soil structural improvement, and plant growth.

### 3.1 Desert microbial resources

The microbial taxa enriched in plant roots, rhizosphere and BSCs constitute the key biological interface connecting plants with desert extreme environments (Table 2). These groups are not only naturally selected survivors of extreme conditions but also act as ecological engineers that directly or indirectly regulate plant-water relationships, and are direct strain resource libraries providing essential functional modules and strain resources for the design of SynComs.

**Table 2:** Selected case studies of desert microbial resources.

Different niches	Representative taxa	Functionality	References
**Bulk soil**	Actinobacteriota, Proteobacteria, and Firmicutes	Drought resistance mechanism (spore formation, EPS secretion, dormancy, synthesis of osmotic regulators (trehalose)	[[72], [73], [74]]
	Ascomycota and Basideomycota, AMF (Glomeromycota)	Form spores, mycorrhizal symbiosis, synthesis of osmotic regulators	[3]
**Rhizosphere soil**	Actinobacteria and Pseudomonas	Key groups maintain the complexity of microbial networks to adapt to stress and improve plant drought resistance	[75]
	*Planococcus*, *Planomicrobium*	Specialized species enhance the adaptability of poplar salt through functional compensation	[76]
**Root**	Pseudomonadota, Cyanobacteriota, Actinomycetota and Gemmatimonadota	Nutrient cycling, secondary metabolite production and environmental adaptation support the resilience of host deserts in arid environments	[77]
	Proteobacteria, Firmicutes, *Pseudomonas* and *Bacillus*	Biofilm formation, quorum sensing and activation of drought resistance pathways enhance wheat drought resistance	[49]
	endophytic fungi (*Neocamarosporium chichastianum*, *Neocamarosporium goegapense* and *Periconia macrospinosa*)	Increased antioxidant enzyme activity and proline content enhanced drought and stress resistance of crops	[50]
**BSCs**	Cyanobacteria	Dormancy, filament winding and secretion of EPS combined with sand, N fixation	[[78], [79], [80]]
	Algae, lichen and moss	Physical properties and binding sand quantity increased significantly with BSCs development	[81]

The bacterial community in desert bulk soils is largely composed of Proteobacteria, Actinobacteria, Bacteroidetes, Firmicutes, and Chloroflexi [[72], [73], [74],^82^]. The rhizosphere, as a microecological hotspot actively regulated by plants, harbors a microbial community with a clear functional orientation. It is dominated by Actinobacteria, Firmicutes (e.g., *Bacillus*) and Proteobacteria (e.g., *Pseudomonas*, *Rhizobium*), and the abundance of Firmicutes and Actinobacteria increases significantly with increasing drought intensity [83]. For example, both the abundant and rare taxa in the rhizosphere of *Artemisia desertorum* are absolutely dominated by Actinobacteria and Proteobacteria, which respectively govern basal metabolic functions and maintain network stability [75]. In the rhizosphere of 11 plant species in the Namib Desert, the microbial communities are also dominated by Proteobacteria, Actinobacteria, and Firmicutes, with *Bacillus* present in the rhizosphere of all plants [84]. Among them, Actinobacteria have become a dominant bacterial taxa in desert soils due to their Gram-positive cell walls and sporulation capacity, extensive metabolic and degradative capabilities, as well as synthesis of secondary metabolites and ultraviolet (UV) repair functions [85,86]; Firmicutes cope with stress by forming endospores to enter dormancy, or produce EPS to improve the local microenvironment, thereby enhancing survival and functionality under harsh conditions [87]; while Proteobacteria contain specific drought-tolerant strains that adapt to resource scarcity through versatile metabolic capacities or acquire drought-tolerant traits via horizontal gene transfer [88].

The fungal community in desert soils is predominantly composed of Ascomycota, Basidiomycota, Glomeromycota, and Zygomycota. These fungi adapt to extreme arid environments by forming spores, synthesizing osmoregulatory substances, and developing hyphal networks [3]. For example, in the rhizosphere of *Pinus sylvestris* var. *mongolica* in sandy lands, ectomycorrhizal fungi (EMF) are dominated by Ascomycota and Basidiomycota, forming ectomycorrhizae with roots to promote plant growth [89]. Furthermore, AMF (Glomeromycota) are widely distributed in desert ecosystems with high diversity, exhibiting significantly greater species richness compared to other ecosystems (e.g., grasslands, farmlands), and are recognized as key microbial groups influencing desert ecosystem functionality [90]. To date, AMF colonization has been detected in desert plants belonging to over 69 families, among which Asteraceae, Poaceae, and Fabaceae are the main plant groups symbiotic with AMF [91]. In desert regions, the isolated AMF strains account for 44.5% of the known AMF strains globally [92]. Among them *Acaulospora* and *Glomus* are the dominant taxa, representing more than 80% of the known AMF species in desert areas [92]. As important symbiotic fungi of plants, both genera can exchange nutrients with plants by forming arbuscules, exhibiting strong adaptability to extreme drought and nutrient-poor environments, and sharing highly consistent functions in facilitating plant nutrient acquisition and enhancing stress tolerance. Numerous inoculation studies have confirmed the universality and effectiveness of these functions. For example, inoculation with *Acaulospora koskei* and *Acaulospora morrowiae* significantly promoted the growth and nutrient uptake of *Parkia platycephala* seedlings under drought stress [93]; in another vegetation restoration study conducted on degraded coastal sand dunes, seedlings of *Guazuma ulmifolia* inoculated with *Acaulospora longula* exhibited the best growth performance [94]. With respect to the *Glomus*, a 10-year field experiment revealed that inoculation with *Funneliformis mosseae* increased the total root length density, root volume, and root surface area of *Hippophae rhamnoides* under drought conditions by 57.21%, 205.87%, and 103.83%, respectively, and enhanced its water uptake from deep soil layers (100-300 cm) by approximately 11% to 12.7%, thereby significantly improving its drought resistance during seasonal water deficiency [95]. A study in the Gurbantünggüt Desert also found that inoculation with *Glomus mosseae* and *Glomus etunicatum* significantly promoted the growth of two ephemeral plants (*Plantago minuta* and *Erodium oxyrhynchum*), increased their N and P uptake, and even increased the seed number of *Plantago minuta* by 50% to 78% [96]. Overall, the roles of AMF in desertified regions are not only reflected in regulating plant physiological functions, enhancing stress tolerance, and promoting vegetation restoration [97], but also in promoting soil aggregate formation, improving soil physicochemical properties, and stabilizing sand dunes, thereby playing important ecological functions in controlling soil erosion and suppressing dust storms [98]. Moreover, under extreme drought conditions, AMF diversity is even the primary factor determining the stability of plant productivity, and its impact may be stronger than that of plant diversity itself [99], highlighting that belowground AMF diversity is a key mechanism for maintaining ecosystem functional stability under extreme climate conditions.

BSCs are complexes composed of bacteria, fungi, algae, lichens, mosses, and their secretions bound to soil gravels, representing a typical land cover in arid regions. They play important role in maintaining soil stability, preventing wind and water erosion, regulating surface water processes, and promoting C and N cycling, and are thus of great significance for sustaining the soil structure and function of arid and semi-arid ecosystems, especially desert ecosystems [81,100]. Among them, cyanobacteria (e.g., *Nostoc*) as photosynthetic prokaryotes, act as pioneer colonizers in the formation of BSCs due to its exceptional drought tolerance and dormancy-activation strategies. Their core ecological functions involve filament entanglement and the secretion EPS to bind sand particles [78,79]. Compared with lichens and mosses, cyanobacteria can not only survive in more arid environments but also expand crust structures more rapidly when water becomes available, thereby swiftly stabilizing the soil surface [79]. Recent research has integrated cellulose nanocrystals (CNCs) with the cyanobacterium *Leptolyngbya ohadii* to develop a novel living material (ICCB). This material not only significantly accelerates the formation of BSCs but also enhances soil resistance to wind erosion and water retention capacity, playing a prominent role in rehabilitating degraded soils in arid areas [101]. Additionally, cyanobacteria can input available N into the soil through biological N fixation, thereby improving soil physicochemical properties and enhancing soil fertility, which is the core driver of initial soil fertility accumulation in bare desert lands [80]. Research indicates that as BSCs succession progression, soil microbial community structure and enzyme activities are progressively ameliorated [102], and sand fixation capacity is also markedly enhanced [81]. In summary, BSCs can create important initial conditions for subsequent plant seed germination and seedling establishment by stabilizing soil structure and enhancing water and nutrient availability, and serve as a vital ecological pioneer layer in the process of desert vegetation restoration [78].

### 3.2 Desert microbial stress resistance mechanisms and ecological effects

In arid environments, microorganisms are susceptible to water deficit, membrane disruption, accumulation of reactive oxygen species (ROS), protein denaturation, and DNA damage. Consequently, these microbial groups have evolved multiple adaptive strategies. Consequently, these microbial groups have evolved multiple adaptive strategies. First, they increase cell wall thickness and strength to reduce water loss and minimize drought-induced cell lysis [103]. Second, under extreme water scarcity, some microbes form dormant structures such as spores or endospores, entering deep dormancy to lower metabolic consumption; these structures are highly resistant to drought, high temperatures and UV, and can recover their activity after brief precipitation events [104]. In addition, microorganisms maintain cellular osmotic balance by accumulating osmoregulatory substances (e.g., proline, betaine, trehalose), thereby preventing protein denaturation and loss of enzyme activity under drought stress [105]. Dryland microorganisms also maintain water and fertilizer by secreting EPS, synthesize cell membranes enriched in unsaturated fatty acids to maintain structural integrity, and adapt to high pH environment by membrane proton and cation pumps [3]. At the same time, they are rich in genes related to C metabolism and DNA repair, thereby enhancing stress resistance and maintaining genome integrity [106]. Under high-salinity conditions, microorganisms accumulate KCl to balance the high osmotic pressure, or alternatively, salt-tolerant strains become enriched and perform their functions. For example, with increasing salt stress, the rhizosphere of *Populus euphratica* (*P*. *euphratica*) significantly enriched halotolerant bacteria *Planococcus* and *Planomicrobium*, which improved the salt resistance of *P. euphratica* [76]. Furthermore, studies have shown that some bacteria (Actinobacteria, Acidobacteria, Proteobacteria, and Cyanobacteria) can utilize atmospheric hydrogen (H_2_) to obtain energy and fix C, independent of photosynthesis [107,108]. This process is considered an important mechanism for desert bacteria to acquire C sources and thereby adapt to environments with prolonged water and nutrient scarcity. In addition to stress resistance and resource acquisition, microorganisms also survive in nutrient-poor desert environments through the sharing of metabolites, i.e., cross-feeding [109].

Additionally, microorganisms create favorable habitats for plants by improving soil structure, facilitating nutrient cycling, and enhancing soil ecological functions. First, microorganisms secrete EPS (including polysaccharides, proteins, and lipids), which act as binding agents between soil particles and stabilize soil structure [105,110]. The glomalin-related soil protein (GRSP) released by AMF further facilitates the formation of soil aggregates and the sequestration of soil organic carbon (SOC) [32], while AMF hyphae transport water to plants via the extracytoplasmic pathway [111]. Second, microorganisms drive soil nutrient cycling and SOM turnover, and concurrently modulate plant hormone signals and the expression of nutrient metabolism-related genes, thereby synergistically improving plant nutrient acquisition [112,113]. Third, rhizosheath—a cylindrical structure formed by microbial compounds (e.g. EPS) cementing sand particles tightly wrapped around the root system—not only protects roots from water deficit but also provides a preferential colonization niche for rhizosphere growth-promoting communities [15,114]. The colonization of these microorganisms increases the content of microbial necromass in the soil. In desertified areas, microbial necromass can contribute up to 40% of the total soil C [115]. Therefore, maintaining living plants and promoting the formation of microbial necromass can gradually establish a C pool protected by mineral binding, aggregate encapsulation, and chemical recalcitrance, thereby enhancing soil ecological functions [15].

Drought is often accompanied by intense light and oxidative stress, leading to excessive accumulation of ROS. Microorganisms alleviate oxidative pressure on plants by forming a rhizosphere oxidative buffer zone via secreting antioxidant enzymes (superoxide dismutase (SOD), catalase (CAT) and peroxidase (POD)) and non-enzymatic antioxidants (e.g., glutathione) that scavenge ROS [116,117]. Additionally, microbes can trigger the plant's endogenous antioxidant defense system, which mitigate oxidative damage and helps sustain normal water uptake and transport. Certain microbes also modify root architecture and modulate stomatal behavior through the synthesis of auxin and abscisic acid, and by suppressing ethylene production, a stress hormone, ultimately improving plant water acquisition efficiency and decreasing water loss via transpiration [118,119]. Beneficial plant microorganisms are also key participants in osmotic regulation, forming the core foundation of plant drought adaptation. Plant-growth-promoting rhizobacteria (PGPR) directly synthesize osmotic regulators to sustain cellular osmotic balance and improve plant drought tolerance [120,121]. Numerous studies indicate that inoculation with PGPR significantly increases the content of these osmotic adjustment substances (e.g., proline, soluble sugar) in plants under drought stress [122,123]. Meanwhile, PGPR also secrete signaling molecules that activate the expression of plant drought response genes and upregulate the biosynthesis of osmoprotectants [105]. In addition, EMF or AMF, as key fungal groups, directly improve water and nutrient use efficiency and stress resistance through mycelial networks and physiological processes such as regulating host antioxidant system and stomatal conductance [[124], [125], [126]]. It is worth noting that under drought, bidirectional metabolic regulation occurs: plants release specific metabolites (e.g., caffeic acid, proline, trehalose, CLE25) to enrich functional microbial taxa, and microbes, in response to plant signals, secrete beneficial metabolites (e.g., pteridic acid, trehalose-6-phosphate, glycerol-3-phosphate, ACC deaminase) that further enhance plant drought tolerance by regulating aquaporin gene expression and strengthening cell membrane stability [83].

In summary, the active adaptation strategies of desert soil microorganisms and their role in mediating host adaptation to environmental stress are key to enhancing plant stress resistance. However, relying on single strains with limited functional capacities makes it difficult to address the complex and variable extreme conditions of desert environments. Therefore, scientifically combining indigenous microorganisms with well-defined genomic backgrounds and specific metabolic functions can achieve synergistic effects at the community level. For instance, a SynCom constructed from PGPR, AMF, and N-fixing cyanobacteria can help early seedling establishment, enhance their drought resistance and soil stability.

## 4 Construction of SynComs

The construction of SynComs aims to achieve directional output of complex functions through rational design and engineering methods, and has both environmental adaptability and controllability. This section describes the design principles and construction methods of SynComs for desert environments.

### 4.1 Design principles

The construction of SynComs primarily adheres to four core principles: ecological adaptability, functional parsimony and modularity, prediction and regulation of interaction networks, and community robustness and stability [40,127]. First, the principle of ecological adaptability requires that the selected microbial members must be able to adapt to the extreme and fluctuating environmental stresses in deserts, such as high temperatures, drought, and salinity. Priority selection of desert indigenous microbial strains can not only reduce the risk of ecological invasion, but also facilitate the colonization of SynComs in the target environment. Second, the rational assembly of communities should be based on functional modules as fundamental units. Microorganisms in a community often form interacting functional clusters, each responsible for specific ecological processes. For example, water regulation module (e.g., AMF and EPS-producing taxa), nutrient cycling module (e.g., N-fixers and P-solubilizers), and stress-mitigation module (e.g., ACC deaminase producing taxa). By integrating these different functional modules, it is possible to construct SynComs that are structurally simple yet functionally diverse. Third, the metabolic interactions (e.g., cross-feeding, competition, antagonism) among functional modules should be predicted to minimize internal competition and metabolic expenditure, optimize resource allocation, and thereby design SynComs with high performance and stability [38]. Finally, through the coordination of inter-strain interactions and the deliberate design of functional redundancy—i.e., employing multiple strains that perform the same function—single-point failure can be avoided. When environmental changes inhibit or eliminate certain strains, other strains can compensate functionally and maintain the overall functional balance.

### 4.2 Construction methodology

To achieve the intended functions, the construction of SynComs typically employs two strategies: “top-down” and “bottom-up” [128]. The top-down approach begins with complex natural microbial communities, from which core functional strains are retained through screening, simplification, and optimization to generate a controllable synthetic consortium [41]. Current top-down methods, mainly comprising enrichment, artificial selection, and directed evolution, satisfy the functional and stability requirements of SynComs design and preserve, to a certain degree, the interaction networks of natural communities [40]. Nevertheless, they are limited in terms of functional regulation, mechanistic interpretation, and experimental reproducibility. By contrast, the bottom-up strategy starts with obtaining individual cultivable strains and their genomic data, reconstructing their metabolic networks, and then employing modeling or analytical tools to guide the rational design and construction of target SynComs [129]. While bottom-up construction offers precise strain control and clear functional division of metabolic tasks, it is constrained by the availability of current strain collections and databases, often resulting in reduced microbial diversity, which may impair the performance of engineered communities under field conditions [40]. To overcome the limitations of both approaches, researchers have begun to integrate them: key functional members are first identified from complex communities, and this is then combined with bottom-up metabolic modeling to predict interspecies interactions and determine optimal strain combinations [38]. For instance, Ruan et al. [130] used herbicides and their degrading bacteria as environmental stress factors to directionally enrich soil microbial communities, isolated key strains, and subsequently applied the SuperCC model to optimize metabolic combinations, ultimately constructing a functionally enhanced SynCom.

Given the extreme complexity and heterogeneity of desert ecosystems, constructing SynComs that effectively enhance seedling water uptake, drought resistance, and survival necessitates an integrated top-down and bottom-up strategy approaches (Fig. 4). The core of this strategy lies in screening and assembling indigenous microorganisms that possess both environmental adaptability and well-defined drought-resistant and growth-promoting functions, while ensuring their ecological efficacy through a multi-tiered verification system. First, in the top-down strain mining phase, the root and rhizosphere microbiomes of pioneer plants (e.g., *Caragana korshinskii*, *Salix psammophila*, *Hedysarum leave*) and BSCs serve as the starting point. By analyzing the dynamic shifts in microbial abundance under drought stress and their topological positions within co-occurrence networks, target taxa that occupy core ecological niches and possess key functional potentials can be identified. These include species carrying genes such as ACC deaminase (*acdS*), EPS synthesis, nitrogenase (*nifH*), and P solubilization-related (*phoD*). Second, during the strain acquisition phase, traditional separation techniques or high-throughput pure-culture platforms are used to directionally isolate and culture target strains [40]. In view of the extreme environmental characteristics of oligotrophic, high salinity and hyperosmotic in desert, dilution medium (to enrich oligotrophic taxa), addition of stress factors (to screen for halotolerant and drought-resistant strains) or specific inhibitors (to isolate slow-growing strains, such as members of Actinobacteria) can be applied to improve the isolation of specific taxonomic groups. Studies on cultivating microorganisms from extreme environments provide a systematic technical framework for this process, significantly expanding the feasibility of exploiting stress-resistant functional microbial resources from desert environments [131,132].

**Fig. 4:**
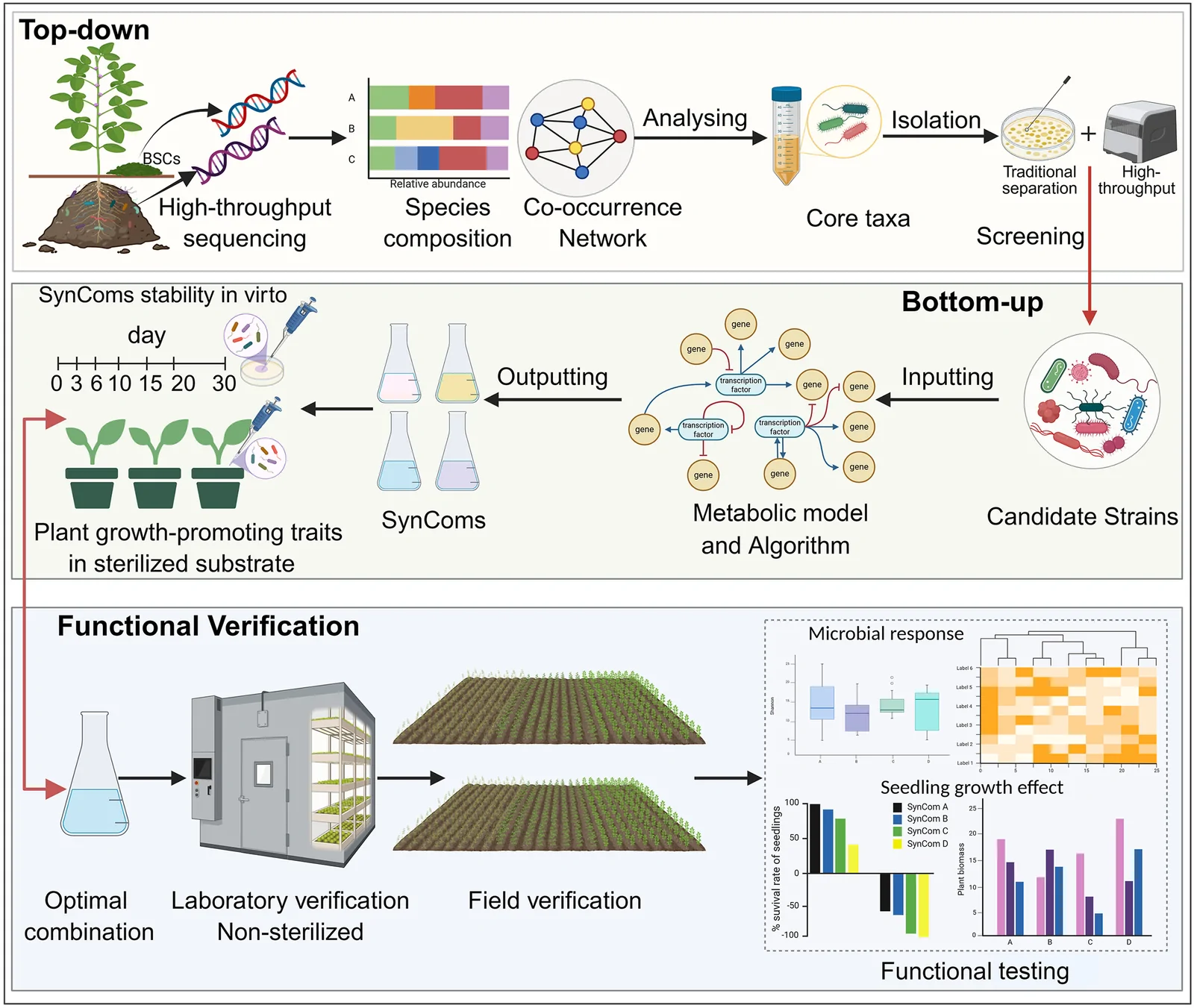
SynComs construction strategies for desert environments. This diagram integrates top-down and bottom-up approaches with functional validation to enable the construction and application of SynComs.

In the bottom-up phase, by integrating genome-scale metabolic models (GSMMs), algorithm optimization, and multi-omics data, the community metabolic network of the obtained culturable strains is subjected to predictive optimization, and keystone species with potential metabolic interaction capabilities are selected to construct the SynComs [133]. Furthermore, the constructed SynComs are subjected to a progressive verification system from laboratory to field. First, the survival state and the ability to promote plant growth were verified in the sterilization system, and the best combination of SynComs were selected. Subsequently, greenhouse experiments simulating drought stress are employed to evaluate the efficacy of the best-performing SynComs in improving seedling WUE, osmotic adjustment, and survival rates. Finally, field trials should be conducted, with a core focus on evaluating their colonization rates, their effects on plant growth, and their long-term impacts on native microbial communities and soil ecological functions.

### 4.3 Artificial intelligence and machine learning for intelligent SynComs design

Beyond conventional construction methods, the application of artificial intelligence (AI) is facilitating a paradigm shift in synthetic biology, from empirical trial-and-error to rational design, and offers a transferable methodological framework for engineering SynComs tailored to desert environments.

At the strain screening level, the rhizosphere microbiome associated with natural “fertile islands” in desert ecosystems serves as a strain resource pool. By integrating AI with conventional microbial screening techniques and employing machine learning (ML) models to refine screening parameters, functional strains capable of coping with desert stresses can be rapidly identified. For instance, the AI-powered Digital Colony Picker (DCP) platform enables high-throughput single-cell screening across multiple phenotypes, accelerating the discovery of elite strains [134]. Similarly, the MiMiC2 automated bioinformatics framework, developed by Thomas Clavel's group and centered on a function-oriented strategy, integrates metagenomic data with isolate genome information, allowing for the rapid identification of target consortia and the design of functionally defined SynCom [135]. Concurrently, AI's capacity to process high-dimensional complex data facilitates the analysis of relationships between microbial traits and environmental factors, thereby enabling the selection of candidate strains optimally adapted to specific habitats. For example, ML models including XGBoost and SVM have been employed to dissect the linkage between microbial communities and crop drought tolerance, enabling the prediction of strain drought resistance, with the optimal model attaining an accuracy of up to 87% [136]. Furthermore, gene editing tools such as CRISPR/Cas9 can be harnessed to engineer metabolic pathways of wild-type probiotics for enhanced drought tolerance, and AI-driven generative models, including deep neural networks and generative adversarial networks, can predict editing outcomes, significantly compressing the timeline for obtaining high-performance strains [137].

At the community design level, ML models combined with GSMMs enable the evaluation of strain metabolic potential, substrate utilization capacity, stress resistance, and interspecific interactions (synergistic or antagonistic), thereby optimizing strain combinations to enhance the stability and functionality of SynComs [137]. For example, the BacterAI platform employs active learning to predict microbial metabolic activity without prior knowledge, accelerating SynComs optimization [138]. The GENIA framework integrates high-throughput isolation, functional screening, whole-genome sequencing, and metabolic network modeling to achieve fully automated workflows from strain selection to community design; the resulting SynCom degraded over 90% of three types of pollutants, with degradation efficiency 1.5-2.2 times higher than that of the optimal single strain [139]. AI can also identify phylogenetically compatible strains to construct cross-kingdom (bacteria-AMF) complementary consortia, which is of great significance for promoting desert vegetation establishment.

Finally, the constructed SynComs are validated in plant systems, where automated high-throughput phenotyping platforms can be used to rapidly assess plant phenotypic traits. The obtained phenotypic data, together with relevant omics data, are then fed back into ML models, establishing a closed loop of data generation, model training, and community optimization, thereby continuously iterating and enhancing the precision of SynComs design [138]. For example, the KAUST team employed the deep learn (DL) model DeepGOMeta to predict genomic functions and identify missing microbial functional units in relatively nutrient-poor desert soils, designed targeted SynCom to fill these gaps, and then simulated stress environments to screen for the most stable combinations that best support plant growth, fully realizing an iterative optimization cycle of functional prediction-community design-dynamic validation [140]. In conclusion, the integration of AI into SynComs design has facilitated the precise development of desert SynComs. Nevertheless, AI applications still confront multifaceted challenges in real-world practice, such as data quality, model interpretability, ecosystem-level stability, and field deployment, and their actual value for ecological restoration remains to be validated through long-term, systematic field experiments.

## 5 The potential role of SynComs in desert seedling establishment

Although indigenous desert microorganisms already possess enhanced stress resistance, their functions are often dispersed among different taxa. In contrast, SynComs, by integrating multiple functionally complementary strains, can synergistically alleviate seedling death caused by hydraulic failure under conditions where basic water availability (e.g., drip irrigation or natural precipitation) is present. Therefore, this chapter analyzes the potential mechanism of SynComs to help seedlings establish and survive from four aspects: improving soil physical structure, promoting WUE and reducing transpiration demand, enhancing physiological resistance and increasing soil nutrient availability (Fig. 5, Table 3).

**Fig. 5:**
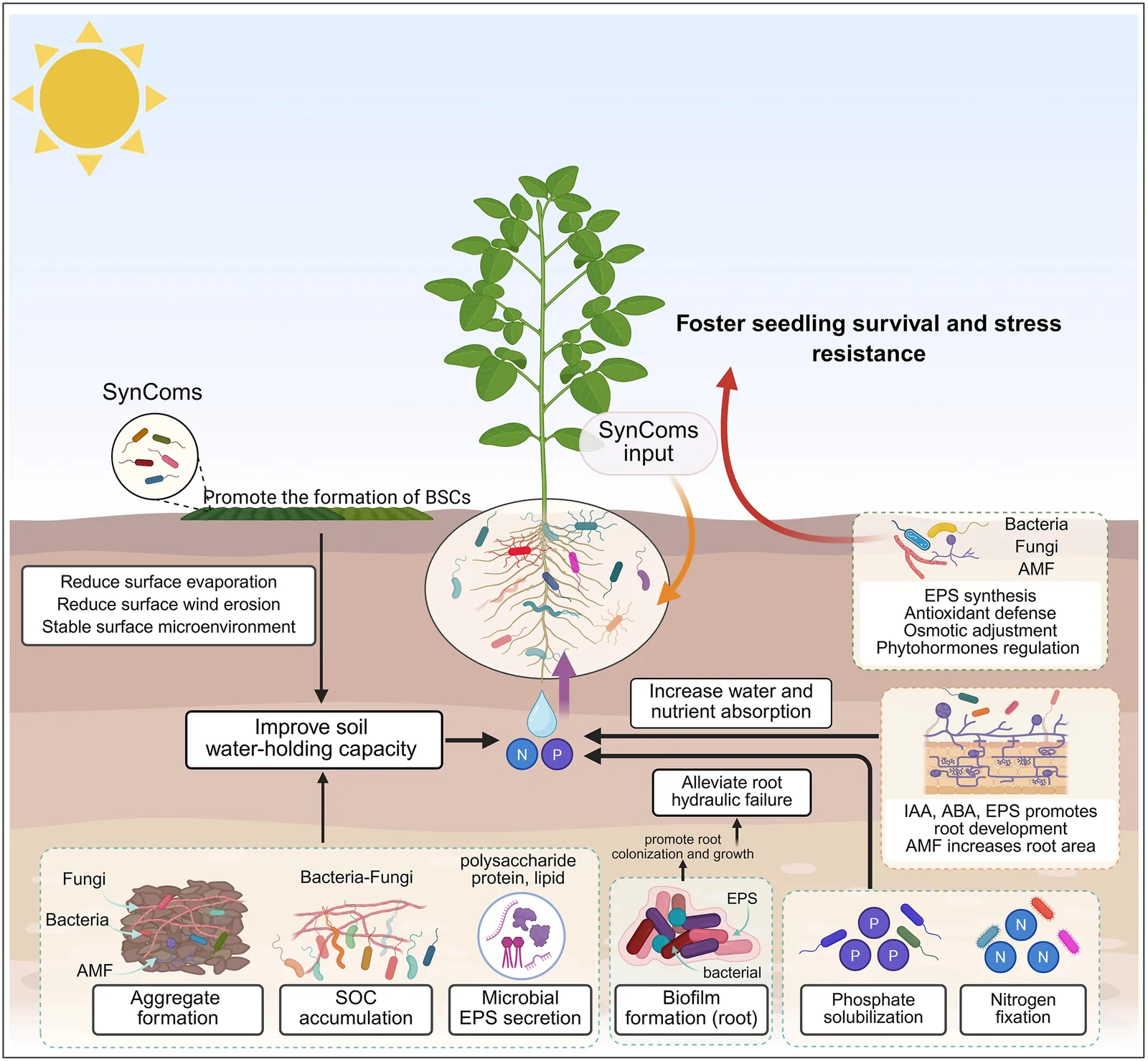
Potential roles of SynComs in desert seedling establishment. This schematic illustrates that introduced SynComs enhance seedling survival through multiple mechanisms, including direct effects on the soil‑plant system (water retention, root promotion, hydraulic failure alleviation, and drought tolerance enhancement) and indirect regulation of the surface microenvironment via the formation of BSCs, which reduces evaporation and stabilizes the surface soil.

**Table 3:** Case studies on the potential roles and mechanisms of SynComs.

Regulatory dimension	SynComs	Mechanisms	Functional implications	Research subject	References
**Improving Soil Structure and Enhancing Water Retention Capacity**	*Rhizobium* sp., *Enterobacter ludwigii*, *Rhodotorula mucilaginosa*; CT205 and SQR-T037	Promote the formation of large soil aggregates	Enhance soil water-holding capacity	Soil	[141]
	*Rhizophagus intraradices* and *Funneliformis mosseae*	Enhancing mycelial networks and GRSP promotes the stability of water-stable aggregates		Soil	[142]
	(*Pseudomonas oryzihabitans*, *Enterobacter ludwigii*, *Brucella intermedia*, *Bacillus LJIY_s*) (bacteria) and *Trichoderma simile* DT36 (fungi)	Promote soil organic carbon accumulation		Soil	[143]
	*Exophiala oligosperma* Zh2 and *Phormidium tenue*	EPS and fungal hyphal networks synergistically promote the formation of biological soil crusts	Form a surface biological water retention barrier to stabilize the rhizosphere microenvironment	BSCs	[144]
	*Bacillus* and *Brevibacterium* and Rhizobiaceae	Stabilize the soil physical structure, supply nutrients, and activate the microenvironment, to accelerate the formation and maturation of artificial blue-green algal crusts		BSCs	[145]
**Enhancing Seedling Water Uptake and Utilization Capacity**	Proteobacteria and 15 other strains	Activate the ethylene signaling pathway to induce lateral root growth	Expand the range and efficiency of water absorption	*Arabidopsis thaliana*	[146]
	*Variovorax* CL14 and *Variovorax* MF160	Degrading the excessively secreted IAA by the bacterial community, and reestablishing the hormone balance in the rhizosphere	Reverse the inhibitory effect of the strain on the root system and enhance water absorption	*Arabidopsis thaliana*	[147]
	*Bacillus* and 16 other strains	Regulate fluid flow and water utilization, reduce turgor loss, and accelerate rehydration recovery	Reduce water consumption and enhance water use efficiency	Maize (crop)	[148]
	*Devosia* sp. ZB163 and AMF	*Devosia* sp. ZB163 promotes the growth of AMF hyphae and enhances root mycorrhizal colonization	Increase plant water use area and nutrient absorption	*Prunella vulgaris*	[149]
	*Acaulospora laevis*(AMF)and *Bacillus subtilus*(PGPR)	Improve root colonization rate, increase IAA and ACC content, and reduce ABA content to improve water use efficiency (12.77%-21.62%)	Expand the dimension of water acquisition and form a fungus-bacteria-plant water channel	Maize (crop)	[150]
**Enhancing Plant Stress Resistance**	*Variovorax beijingensis* and 6 other strains	Enhance antioxidant enzyme systems, photosynthetic efficiency and biomass (50.10%–76.40%)	Enhance the drought resistance of *Astragalus mongholicus*	*Astragalus mongholicus*	[151]
**Enhancing Plant Stress Resistance**	*Bacillus*, *Protomicromonospora* and *Streptomyces*	Regulate endogenous hormones (increase ABA content) and accumulate osmoregulatory substances (increase Pro content)	Enhance the drought resistance of desert forage	*Neopallasia pectinata*	[152]
	*Pseudomonas* CM-9 and 4 other strains	Enhance biofilm formation and EPS production, improve antioxidant defense, enhance osmotic regulation and increase biomass (50%)	Strains from *Caragana korshinskii* enhance drought resistance in wheat	*Caragana korshinskii*, wheat	[49]
	Five SynCom (5,7,8,9 and 10)	Reduce oxidative stress, regulate osmotic substances, and balance ions	Enhancing salt tolerance of Poplar 84K	*Populus euphratica*, Populus 84K	[120]
	Six SynComs (SCA, SCB, SCC, SCD, SCE, and SCF)	Regulate osmotic pressure, secrete antibiotics	Enhance the resilience of poplar against drought, salt stress, and diseases	Populus 84K	[153]
	SynComs C (SA403, SA087, SA187, SA436, SA444)	Up-regulate the expression of salt stress-related genes and reduce the Na^+^/K^+^ ratio	Microorganisms from the desert rhizosphere enhance tomato resistance to stress	*Indigofera argentea*, tomato	[48]
	SynComs (*Flavobacterium* and 8 other strains) and EMF (*Tuber*)	Up-regulate genes involved in butyrate metabolism and DNA replication in EMF, and alleviate the inhibitory effect of soil metabolites on EMF	Promote the growth of poplar under drought conditions	poplar	[154]
**Improving soil fertility**	*Arthrobacter*, *Bacillus*, *Acinetobacter*, *Pseudomonas*, and *Mixta*	Enrich beneficial bacteria and enhance biodiversity, while increasing the complexity of the bacterial network	Enhance the nutrient function of desert soil	Soil	[155]
	*Arthrobacter*, *Bacillus*, *Acinetobacter*, *Pseudomonas*, and *Mixta*	Enhance the abundance of functional genes related to C, N and P cycles, as well as soil enzyme activity, and reshape the bacterial community structure	Enhance the soil multifunctionality of desert	Soil	[156]
	*Bacillus subtilis* and *Bradyrhizobium japonicum*	Enhance the activities of soil DHA (269%) and ALP (149%), thereby improving the conversion efficiency and storage capacity of available N, P, and K in the soil.	Improve soil fertility	*Vigna mungo* L.	[157]

Note:The cases listed above encompass both desert and non-desert plant species, with some experiments conducted under drought or salinity stress. Although the host species and environmental conditions differ from those of natural desert ecosystems, these studies still provide valuable insights and serve as useful references for the application of SynComs in desert seedling establishment.

### 5.1 Improving soil structure and water retention capacity

Microorganisms directly influence soil water retention and infiltration capacity by modifying soil structure. The core of soil structure lies in aggregates, and microorganisms primarily affect the formation and stability of aggregates through physical entanglement and chemical cementation. Fungi are adept at mediating the surface polarity of soil particles through hyphal entanglement and the secretion of hydrophobins, thereby dominating the formation of macroaggregates [158]. EPS secreted by bacteria, fungi, and cyanobacteria is one of the main factors contributing to aggregate formation [110,159]. Furthermore, interactions among members of SynComs are more conducive to aggregate formation. For example, after treatment with unicellular microorganisms (*Rhizobium* sp., *Enterobacter ludwigii* and *Rhodotorula mucilaginosa*) and with a mixture of unicellular and filamentous microorganisms ((*Steptomyces albospinus*) CT205 and (*Trichoderma harzianum*) SQR-T037), the soil aggregate content increased significantly, reaching 7.0 and 7.5 times that of CK, respectively. This was mainly attributed to EPS secretion by the unicellular microorganisms and hyphal entanglement by the filamentous microorganisms [141]. In addition to promoting aggregate formation, EPS itself can enhance the water-holding capacity of sandy soils, reduce hydraulic conductivity, and slow evaporation rate [80,110]. For instance, applying EPS produced by strain *Kocuria carniphila* AUMC B-453 to sandy soil can improve soil water retention capacity and aggregation properties, and promote plant growth under low irrigation conditions [160]. EPS also mediates bacterial cell adhesion and aggregation, providing a three-dimensional scaffold for biofilm formation and facilitating biofilm colonization on surfaces [161]. Although the extent of biofilm formation in soil remains debated [162], studies have confirmed that many desert PGPR (e.g., *Pseudomonas*) are capable of forming biofilms in the rhizosphere [49,163], which enhances root colonization and growth, and sustains hydraulic continuity by creating a hydrophilic buffer layer at the root-soil interface, delaying the hydraulic failure induced by drought. Furthermore, biofilm-forming SynComs have been shown to outperform single strains in improving plant drought survival [164,165]. In addition, AMF contribute to soil aggregation by secreting GRSP and forming a hyphal network that acts as a skeletal framework, binding dispersed sand, silt, and clay particles into macroaggregates [166]. Research indicates that AMF presence can double the efficiency of macroaggregate formation and significantly retard aggregate disintegration [166]. This enhancement arises from the dual stabilizing effect of AMF through increased hyphal density and GRSP production, as evidenced by studies inoculating with *Rhizophagus intraradices* and *Funneliformis mosseae* [142]. Therefore, the construction of SynComs that can efficiently produce EPS is crucial for improving soil structure, enhancing sand soil water holding capacity, and increasing plant drought resistance.

Increasing SOC and reducing its loss are key to improving soil physical structure and strengthening water and nutrient retention [167]. SynComs constructed based on photoautotrophic bacteria can utilize cyanobacteria or microalgae as core C-fixing carriers, forming functionally synergistic C cycling networks with heterotrophic bacteria [168]. In this system, C-fixing functional bacteria are responsible for converting atmospheric CO_2_ into organic substances such as sugars and phenols, while organic matter-decomposing communities such as actinomycetes and fungi promote the transformation of SOC into stable forms through degradation, and gradually drive the accumulation of SOC. In addition, EPS secreted by microorganisms also constitutes a significant fraction of the soil C pool. EPS contributes to SOC mainly through microbial-derived direct C input, promotion of aggregate formation, and interaction with sand mineral particles to form organo-mineral complexes, which are critical for the accumulation and stabilization of SOC [169,170]. Research has confirmed that cross-kingdom SynComs constructed with PGPR and *Trichoderma* fungi can significantly improve SOC sequestration efficiency [143]. More critically, increased SOC content substantially improves the hydrological properties of sandy soils. As SOC levels rise, the water-holding capacity of all soil types tends to increase, with the most pronounced gains occurring in sandy and silty soils [171].

As the surface barrier of desert, BSCs play a critical role in reducing surface evaporation, mitigating wind erosion, and stabilizing the soil surface. Current studies have verified the potential of SynComs to drive the formation of BSCs from different levels. Early research demonstrated that inoculation with the indigenous cyanobacterium *Phormidium* from the Qinghai-Tibet Plateau successfully induced artificial BSCs formation [172]. In a subsequent study, Zhou et al. [144] co-inoculated the high EPS-producing fungus *Exophiala oligosperma* Zh2—isolated from lichen crusts in the Kubuqi Desert—with the cyanobacterium *Phormidium tenue*, which significantly promoted BSCs development, with increases in crust biomass, microbial biomass, nutrient content, enzyme activities, and SOM accumulation [144]. Additional studies have further demonstrated the synergistic actions of multispecies consortia. A SynCom constructed from three key functional strains isolated from natural cyanobacterial crusts, EPS-producing, siderophore-producing, and N-fixing bacteria, substantially facilitated the formation of artificial cyanobacterial crusts [145]. Mechanistic analysis indicated that these three microbial groups perform temporally and progressively differentiated roles during BSCs formation: EPS-producing bacteria stabilize soil particles and improve the microenvironment in the early stages through EPS secretion, laying the foundation for cyanobacterial colonization; N-fixing bacteria establish mutualistic relationships with cyanobacteria by providing N sources during the middle to late stages of crust development, significantly enhancing biomass; and siderophore-producing bacteria indirectly enhance the functional activity of the entire microbial community by increasing the bioavailability of trace elements. These findings suggest that SynComs facilitate the formation of BSCs; once crusts are established, microbial metabolism, nutrient uptake, and SOM turnover are quickly stimulated, thereby generating favorable soil conditions for subsequent plant colonization.

### 5.2 Enhancing seedling water uptake and utilization capacity

Establishing beneficial interactions with microorganisms is a key strategy for plants to regulate root development and actively adapt to arid environments. Rhizosphere bacteria can reshape root architecture by secreting or degrading phytohormones, thereby expanding the interface for water acquisition. Research demonstrated that SynCom composed of 16 strains systemically induce lateral root proliferation in *Arabidopsis* by activating the ethylene signaling pathway within roots [146]. Among 185 strains of bacteria SynCom, *Variovorax* is the key genus to reverse the inhibition of root phenotypes by bacterial flora, revealing a core mechanism by which microbial interactions regulate root development [147]. Furthermore, key bacterial strains isolated from the roots of the desert plant *Caragana korshinskii* were used to construct a 15-member SynCom, which maintained drought resistance potential in non-sterile soil by optimizing wheat root structure as well as water and nutrient absorption [49].

In addition to stimulating root development, certain microorganisms can directly enhance plant WUE through two complementary strategies: enhancing water acquisition and reducing water loss. On the acquisition side, AMF extend their hyphal networks expanding the water capture range. Moreover, signaling molecules released by AMF can recruit beneficial bacteria to form a functional consortium in the hyphosphere, further facilitating water transport [173]. On the conservation side, *Streptomyces* sp. HM35 secretes pteridic acid, which activates the plant ABA signaling pathway and inhibits stomatal opening, thereby increasing leaf relative water content by 10-15% under drought conditions [83,174]. Additionally, VOCs produced by various beneficial microorganisms can induce stomatal closure, thereby reducing ineffective transpiration losses [175]. A SynCom composed of 17 bacterial strains has been shown to enable maize to achieve more regulated transpiration, reduced water loss, and enhanced water retention under drought stress, while also accelerating recovery after rewatering by minimizing turgor loss [148].

Additionally, cross-kingdom SynComs constructed with bacteria and AMF exhibit even greater synergistic potential. For instance, co-inoculation of the hyphosphere-isolated strain *Devosia* sp. ZB163 with AMF not only increased plant biomass but also significantly improved AMF colonization and functionality [149]. Under moderate to severe drought, combined inoculation with *Acaulospora laevis* (AMF) and *Bacillus subtilus* (PGPR) enhanced root colonization rates by 104.6%-113.2%, root hydraulic conductivity by 1.9%–1.4%, and WUE by 21.62%-12.77% [150]. Collectively, the rational design of cross-kingdom SynComs comprising PGPR and AMF can synergistically boost water uptake while curtailing water loss, representing a critical microbial strategy for enhancing the survival of desert seedlings.

### 5.3 Enhancing plant stress resistance

Microbial communities are key drivers in alleviating plant drought stress, with their effects spanning multiple levels from molecular responses to physiological regulation. Microbial communities are key drivers in alleviating plant drought stress, with their effects spanning multiple levels from molecular responses to physiological regulation, including the mitigation of oxidative stress, regulation of phytohormones, and expression of drought-resistance genes. Research demonstrated that a SynCom constructed from seven bacterial strains, when inoculated into *Astragalus mongholicus* (*A. mongholicus*), significantly increased the activities of antioxidant enzymes (CAT, POD) and reduced malondialdehyde (MDA) content, thereby alleviating oxidative damage and enhancing photosynthetic efficiency. It also increased the production of IAA and EPS, resulting in a 50.10%–76.40% increase in the biomass of *A. mongholicus* seedlings under drought stress [151]. Another study on the desert forage *Neopallasia pectinata* revealed that SynCom enhance drought tolerance by regulating endogenous phytohormones (ABA) and osmolytes (Proline) [152]. Furthermore, a simplified SynCom5, constructed from root core microbes of the desert plant *Caragana korshinskii*, significantly enhances drought tolerance in wheat crops through multiple mechanisms: at the microbial community level, SynCom5 enhances community biofilm formation and EPS production through AI-2-mediated quorum sensing, improving rhizosphere colonization efficiency; at the plant signaling level, SynCom5 activates the plant MAPK and jasmonic acid (JA) signaling pathways, thereby enhancing antioxidant defense and stress response capacity; at the plant metabolic level, it reprograms carbohydrate metabolism to support energy homeostasis and osmotic adjustment [49]. Notably, the synergistic effects of SynComs exhibit stress-intensity dependence. Research shows that under mild and moderate drought stress, SynComs is significantly superior to single strains in promoting biomass accumulation and inducing stress-resistant substance production in desert forage plants. However, under extreme drought conditions, no significant differences were observed between the effects of single strains and SynCom on plant biomass [152]. Similarly, under moderate drought stress, SynCom inoculation significantly enhanced the drought resistance of *Astragalus mongholicus* [151]. This stress-intensity dependence can be understood from three aspects. First, soil microbial diversity and ecological functions are subject to drought thresholds, and SynComs is no exception. Once soil water potential decreases beyond a critical point, community colonization rates decline, accompanied by a concomitant loss of functionality [176]. Second, under extreme drought, plants prioritize their own survival by reducing photosynthetic C allocation to the rhizosphere, thereby limiting the C supply that beneficial microbial consortia rely on for metabolic maintenance and weakening the mutualistic interactions between the microbial community and the host plant [104]. Third, prolonged drought stress drives a life-history strategy shift in the microbial community from fast growth toward survival priority, a transition that reduces the capacity of the microbial community to promote nutrient cycling and support plant productivity [177,178]. Therefore, in practical applications, it is essential to fully consider the critical environmental thresholds for maintaining the growth-promoting functions, colonization ability, or community stability of SynComs. Accordingly, under severe drought stress, it may be necessary to introduce more extremely tolerant functional strains or to supplement with other protective strategies (e.g., water-saving irrigation or water-retaining agents).

SynComs can also effectively improve plant tolerance to saline-alkaline sandy soils. Taking *Populus euphratica* as an example, it has long survived in high-salinity soils (>15 g kg^−1^), and its rhizosphere microbes are regarded as a natural gene pool for salt tolerance. SynComs constructed from this pool enhance the salt resistance of poplar 84K: on the one hand, they directly reduce oxidative stress, regulate osmotic substances, and maintain ion balance; on the other hand, they enrich salt-tolerant bacteria such as *Phenylobacterium* and *Sphingomonas*, which show significant positive correlations with root length, chlorophyll content, and POD activity [120]. This indicates that SynComs indirectly improve plant salt tolerance by reshaping the rhizosphere community*.* SynComs constructed from the rhizosphere microbiome of poplar under drought, salt stress, and pathogen infection help plants cope with the corresponding environmental stresses through pathways such as osmotic adjustment and antibiotic secretion [153]. Another study showed that Co-inoculation of desert halophyte rhizobacteria *Bacillus pumilus* HR and *Zhihengliuella halotolerans* SB improved *Nitraria schoberi* seedling tolerance to salinity and dust through enhanced physiological and nutritional condition [179]. Additionally, research on the desert plant *Indigofera* has shown that its survival is highly dependent on native rhizosphere microorganisms: in sterilized Jizan soil, *Indigofera* fails to complete its life cycle. A five-strain SynCom from their roots upregulated salt-stress genes expression and reduced the Na^+^/K^+^ ratio, conferring high salt tolerance to tomato under non-sterile conditions and demonstrating cross-host potential of functional consortia [48]. Similarly, three endophytic fungal strains isolated from desert plant roots significantly increased chlorophyll concentrations and promoted growth in tomato and cucumber under both drought and salt stress [50].

In addition, cross-kingdom SynComs hold considerable promise for improving the stress tolerance of desert vegetation. A study showed that inoculation with a SynCom comprising nine key bacterial strains, sourced from the mycorrhizosphere of poplar in arid environments, significantly increased the EMF colonization rate from 18.83% (with Tuber alone) to 33.74%, via a dual mechanism involving direct physical contact and the alleviation of soil metabolic inhibition; this was also accompanied by a marked promotion of host plant growth [154]. Given that EMF enhance plant drought tolerance by optimizing root architecture and improving physiological performance [180,181], this finding offers a viable strategy for leveraging SynComs in combination with EMF to improve afforestation success in desert regions (e.g., poplar and *Pinus sylvestris* var. *Mongolica*). However, dominant desert plants such as *Caragana korshinskii, Salix psammophila*, and *Hedysarum scoparium* primarily associate with AMF rather than EMF, directing our attention toward AMF-based cross-kingdom consortia. Single AMF inoculation with has been shown to promote nutrient uptake and alleviate drought stress in the desert ephemeral *Plantago minuta* [182,183]. Meanwhile, it is recognized that AMF and PGPR are functionally complementary and ecologically synergistic, and their co-application may confer superior stress tolerance compared with single inoculants [184]. To date, however, most evidence supporting this synergy comes from crop systems such as maize and wheat [148,185], where the complementary roles, bacteria driving N/P mobilization and phytohormonal modulation, and AMF expanding water uptake and nutrient translocation, offer valuable lessons for desert applications. Accordingly, the selection of functional strains from desert habitats and their assembly with native dominant AMF into cross-kingdom SynComs holds promise for improving seedling establishment and stress adaptability through multiple avenues, including root system development, water supply, nutrient provisioning, and stress alleviation.

### 5.4 Improving soil fertility

Through synergistic interactions among their members, SynComs are involved in the biogeochemical cycling of key elements such as C, N, and P in soil, thereby enhancing soil fertility and supplying seedlings with a continuous nutrient source. In desertification land restoration, SynComs enhance nutrient availability by increasing soil bacterial diversity and enriching beneficial microbial taxa [155]. A SynCom assembled from in situ probiotics was further shown to increase the abundance of functional genes associated with C, N, and P cycling, along with soil enzyme activities, thereby promoting overall soil multifunctionality (SMF) [156]. Additionally, a SynCom consisting of *Bacillus subtilis* and *Bradyrhizobium japonicum*, when applied in combination with organic amendments, synergistically increased soil dehydrogenase (DHA) and alkaline phosphatase (ALP) activities [157]. These findings collectively validate the practical efficacy of SynComs in activating soil nutrient pools. At the functional level, different SynComs drive nutrient cycling through division of labor and synergistic interactions: N-fixing SynComs expand soil N pools by enhancing nitrogenase activity [186]; P-solubilizing SynComs convert recalcitrant P (e.g., calcium phosphate, iron phosphate, and aluminum phosphate) into plant-available through acidification, chelation, and exchange reactions [187]; and C-fixing SynComs gradually accumulate SOC through two complementary pathways—microbial C fixation and microbe-mediated plant organic C input [188].

Beyond bacteria, AMF also play a critical role in the maintenance of soil functions. For instance, despite warming-induced reductions in soil moisture, the enhanced complexity of the AMF network associated with *Caragana korshinskii* sustained mycorrhizal colonization at relatively high levels (82%–198%), thereby increasing SMF by 43%–228%. Conversely, the collapse of the AMF network in *Artemisia ordosica* led to a decline in colonization rates and a corresponding decrease in SMF [189]. Synergistic studies on AMF and rhizobacteria in drylands indicate that the combined inoculation of AMF and PGPR outperforms single inoculations: compared with the control, AMF, PGPR, and co-inoculation increased SOC by 39%, 38%, and 47%, respectively [190]. Under the mounting stress of climate change, how to effectively integrate the functional plasticity of bacteria with the network stability of AMF, and thereby construct a plant-AMF-bacteria tripartite symbiosis system that could more robustly improve desert soil quality and ecosystem resilience, remains an open question that warrants systematic investigation [184,191,192].

In summary, the bidirectional feedback between plants and microbes—where plants actively recruit beneficial rhizosphere microbes, which in turn enhance host stress tolerance—is a fundamental mechanism maintaining the stability of desert ecosystems [2]. The above studies indicate that SynComs can support vegetation survival in arid environments through multiple complementary pathways, including soil structure improvement, root system optimization, stress tolerance enhancement, and nutrient cycling stimulation. Consequently, reintroducing functionally defined microorganisms from desert ecological niches into degraded habitats, where they can interact with native communities to facilitate seedling establishment, represents an ecological restoration strategy worthy of further exploration. Critically, successfully established plants continuously supply organic matter to the soil via litter, root exudates, and rhizodeposits, thereby recruiting more beneficial microbial taxa and enhancing SMF (e.g., structural stability, nutrient turnover, and C sequestration). This establishes a positive feedback loop that provides sustained momentum for the long-term recovery of desert ecosystems (Fig. 6).

**Fig. 6:**
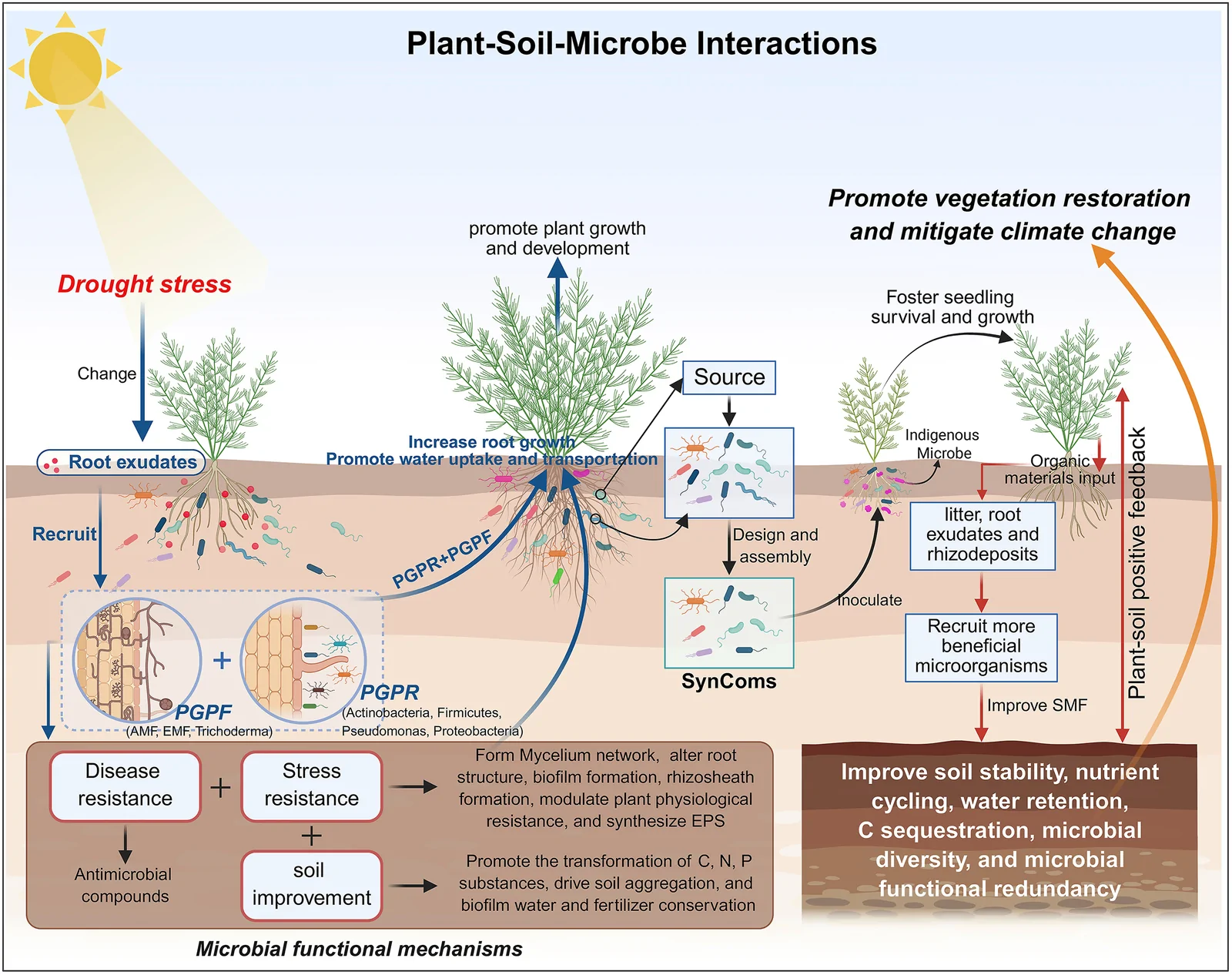
Conceptual framework for integrating SynComs into the plant–soil–microbe positive feedback loop to maintain desert ecosystem stability. SynComs are constructed from functionally specialized microorganisms enriched in vegetation under stress conditions, and are inoculated into the seedling rhizosphere to promote survival and growth. They in turn recruit more beneficial microorganisms to enhance soil functionality and plant stress tolerance, establishing a positive feedback loop that ultimately facilitates vegetation restoration and contributes to climate change mitigation. PGPF, plant growth-promoting fungi; PGPR, plant growth-promoting rhizobacteria; SMF, soil multifunctionality.

## 6 Challenges and prospects

Although SynComs demonstrate significant potential in the aforementioned applications, achieving their stable and predictable functional output to help seedling establishment in complex and variable desert environments still faces numerous challenges. These challenges include functional stability and environmental adaptability, limitations of key technologies, and ecological practicality.

### 6.1 Functional stability

To facilitate control and study, SynComs are usually simplified communities composed of several to dozens of strains [41]. However, desert vegetation establishment depends on the collaboration of soil improvement, plant root development and physiological regulation. This simplified construction paradigm may lead to the loss of key or bridge species essential for realizing complex synergies, and may also weaken key ecological functions such as community stability, functional redundancy, anti-interference and niche occupation by reducing species diversity [164,193]. Moreover, current research has largely focused on high-abundance microbial taxa, while studies on desert ecosystems increasingly reveal that many auxiliary and low-abundance rare also play disproportionately important roles in maintaining soil health and functional stability [75,194,195]. Therefore, designing SynComs entails a critical trade-off between taxonomic diversity and ecological functionality—while high diversity can boost functional performance, it diminishes controllability and scalability, whereas excessive simplification may undermine community stability [193,196]. To construct functionally stable SynComs, we propose the following strategies: (1) Use microbial co-occurrence network tools to prioritize strain combinations with potential synergistic interactions rather than selecting solely based on taxonomic status. Special attention should be given to the dominant taxa driving core functions, rare taxa providing key auxiliary support model, so as to preserve the advantages of asymmetric interactions in natural ecosystems; (2) Maintaining moderate redundancy, combined with rational ecological and metabolic design, equips the community with backup and compensatory capacities to withstand environmental fluctuations and internal perturbations; (3) Construct spatially structured collaborative networks by using carrier immobilization (e.g., biochar, hydrogels) or inducing biofilm formation, thereby achieving spatial compartmentalization and material exchange among strains, promoting division of labor and quorum sensing communication, and thus enhancing disturbance resistance; (4) Introduce dynamic adaptation mechanisms by integrating dormancy-reactivation strategies (e.g., sporulation and germination) and phenotypic plasticity, enabling the community to reduce metabolism during drought periods and rapidly recover activity upon precipitation pulses, thereby maintaining long-term ecological resilience.

### 6.2 Technological challenges

The construction of SynComs still faces technical challenges ranging from strain acquisition and community design to large-scale production, which together constrain SynComs from the laboratory to the field. Firstly, regarding strain resource acquisition, due to highly specialized ecological niches and limitations of traditional cultivation methods, the vast majority of desert microorganisms remain uncultured and uncharacterized [197]. Although culture-independent techniques such as metagenomics can reveal potential genetic resources, they lack directional screening and empirical verification methods for target functions [198,199]. In contrast, cultivation-dependent approaches can not only obtain complete genomic information but also provide an irreplaceable basis for microbial physiology and enzymology research and the development of genetic operating systems. However, traditional cultivation methods often fail to successfully culture currently uncultured microorganisms [200]. Therefore, how to break through the bottleneck of culturability of desert microorganisms is still the primary obstacle to constructing efficient SynComs. Currently, isolation methods vary significantly among different laboratories, such as the use of pretreatment techniques like ultrasonic treatment and UV irradiation [201], which makes it difficult to exchange and standardize the strain resources and increases the difficulty of effective comparison between methods. Secondly, at the community design level, microbial interactions are complex and dynamic. Existing models struggle to predict the higher-order interactions and functions involving more than three species, leading to designed SynComs often failing to maintain stable expected functions after introduction into complex environments due to disrupted internal community interactions [202,203]. Exploring higher-order interaction mechanisms and constructing SynComs that better reflect real ecological scenarios has become a key direction for overcoming this bottleneck. Meanwhile, conventional metagenomic and amplicon sequencing only provide information on the relative abundance of species and cannot quantify the absolute abundance of keystone functional microorganisms, resulting in a lack of precise basis for determining strain ratios during SynComs construction [40]. Most current studies employ simplified strategies with equal ratio assembly, which fails to reflect the true abundance relationships of members in natural communities and may deviate from expectations in practical applications [204]. Finally, at the level of large-scale production and field delivery, during the industrial fermentation amplification of laboratory-optimized SynComs, differences in growth rates and metabolic requirements among strains may impede the growth of certain members and disrupt the original balanced state of the community [205]. Furthermore, microbial inoculants require suitable carriers. How to select liquid and solid delivery carriers to enable the introduced strains to survive effectively, colonize stably and exert corresponding functions upon initial contact with sandy soil and after successive drought periods remains an urgent challenge in the industrialization process [206].

In the future, enhancing the culturability of functional desert microbial strains is fundamental for constructing efficient SynComs. Culturomics-based metagenomics strategy, integrating culturomics and metagenomics (full-length 16S rRNA amplicon and shotgun sequencing), can significantly improve the isolation efficiency and diversity coverage of microorganisms in desert environments [131,207]. Based on this, high-throughput screening techniques should be combined with new microbial isolation and cultivation methods, such as single-cell sorting, microfluidics cultivation, and membrane diffusion cultivation, to expand the strain resource library [133,200]. Furthermore, to enhance the functional robustness of SynComs, the prediction and optimization of microbial interactions can be approached from three aspects: (1) using AL-assisted network analysis tools to screen phylogenetically compatible strains from the local microbiome [208]; (2) designing strain combinations with metabolic complementarity based on GSMMs and ML [138]; and (3) dynamically optimizing strain ratios through computational modeling to predict their effects on community stability and functional output [133]. Finally, the development of microbial formulation technologies, such as soil inoculation, seed coating, and hydrogel-based protective delivery, is essential to ensure the efficient and stable transport of microorganisms to the target plant rhizosphere [209,210]. For example, a polymer hydrogel (PMH) used to load and protect endophytic PGPR not only enhanced their growth-promoting efficiency but also facilitated their effective adaptation to the environment [211].

### 6.3 Ecological effectiveness and regulatory challenges

Owing to unpredictable responses to physicochemical factors (e.g., temperature, pH, nutrients), there is high variability in the survival, colonization, and functional expression of SynComs in field environments [212]. Secondly, the introduction of SynComs may pose potential ecological risks. Released SynComs might display unintended persistence or proliferation, which may displace or inhibit native microbes via resource and niche competition. In some cases, this could lead to soil nutrient depletion or functional decline of beneficial indigenous microbiota. Thirdly, current research is mostly conducted at the laboratory or small-scale field control stage, focusing primarily on crop yield enhancement, soil health improvement, and disease control [17,153], while neglecting possible legacy effects of the introduced SynComs (e.g., horizontal gene transfer, retained mutations, or establishment of novel homeostasis) [213]. An additional challenge is to verify the direct causal relationship between SynComs and plant growth or traits. Finally, unlike other ecosystems, relying solely on SynComs in deserts is still insufficient to achieve effective seedling establishment on moving dunes; integration with physical sand stabilization (e.g., BSCs, straw checkerboard), water management (e.g., drip irrigation), and vegetation selection (e.g., native psammophytes) is necessary.

Therefore, indigenous core microorganisms should be selected as chassis strains to leverage their inherent environmental adaptability [133]. Meanwhile, carriers such as biochar and hydrogels can be used to encapsulate synthetic communities, creating a locally stable microenvironment that buffers against drastic fluctuations in physicochemical factors. Furthermore, CRISPR-Cas9 gene editing technology can be employed to knock down negative regulatory genes, overexpress or introduce exogenous stress-resistance genes, thereby directionally enhancing strain colonization ability, drought tolerance, and water regulation functions [214,215]. High-throughput metagenomic sequencing combined with fluorescence labeling can be employed to monitor community dynamics [216], while the integration of transcriptomics and metabolomics allows systematic analysis of the interaction patterns, functional metabolic pathways, and long-term ecological evolution between synthetic and native microbial communities [217], thereby elucidating the legacy ecological effects of the consortia and reducing potential ecological risks. Finally, given the extreme nature of desert environments, region-specific application strategies should be adopted according to aridity level: in arid regions, starter irrigation or superabsorbent polymers should be co-applied, with the core value of SynComs being the extension of irrigation intervals and reduction of watering frequency; in semi-arid regions, inoculation can be aligned with natural precipitation windows; on stabilized sandy lands or in areas with condensate water, extremely drought-tolerant strains (e.g., cyanobacteria) can be selected to partially dispense with irrigation, or natural precipitation can be used to activate microbial activity. In summary, regulating soil moisture according to actual needs is a key prerequisite for SynComs to exert their functions, with the goal of enabling the soil to gradually acquire the capacity for fertility building, water retention, and growth promotion. This approach not only saves water but also sustainably improves soil quality, representing an important direction for vegetation restoration in arid regions.

### 6.4 A research framework of SynComs for seedling water-nutrient coupling constraints

Community stability, technical bottlenecks, and ecological risks represent the primary barriers to translating SynComs from laboratory research to field application. Nevertheless, the effectiveness of SynComs in alleviating the dual stresses of hydraulic failure and nutrient limitation during seedling establishment hinges on their ability to precisely regulate seedling water and nutrient use. To this end, we propose a rhizosphere-root synergistic regulation framework, which positions the functional design of SynComs across three levels. (1) Rhizosphere interface regulation: multi-strain cooperation secretes EPS to build a rhizosphere biofilm matrix, improving sandy soil aggregation and water-holding capacity, thereby reducing negative pressure accumulation for root water uptake [165]. Meanwhile, the SynComs achieves functional complementarity through P solubilization, N fixation, and siderophore production, synergistically activating soil nutrients [157]. This breaks the vicious cycle of water deficit inhibiting nutrient mineralization, while nutrient deficiency weakens root osmotic adjustment, and reestablishes a coordinated supply of water and nutrients in the rhizosphere. (2) Root architecture remodeling: through the dual pathways of exogenous phytohormone secretion (IAA, GA, ET, CTK, etc.) and modulation of endogenous plant hormone signaling, the strains promote lateral root and root hair development, thereby expanding the root absorption surface area for both water and nutrients [16]. (3) Through synergistic interactions, microbial consortia promote the accumulation of osmolytes and modulate the differential expression of aquaporin genes, which collectively improve plant stress tolerance and water transport efficiency [49].

Based on the above framework, future research should focus on the following key pathways: (1) AI-enabled “hydraulic-nutrient” dual-oriented strain screening. On the basis of conventional functional screening (P solubilization, N fixation, etc.), additional hydraulic-related constraints (EPS secretion, phytohormone synthesis) should be incorporated. ML can then be applied to prescreen the native strain library, enabling rapid identification of candidate consortia with high synergy and high stability. (2) Dynamic stress simulation and optimization of application strategies. AI models should be used to simulate community succession dynamics and metabolic fluxes under various drought-nutrient gradient combinations, predicting the optimal timing and dosage for SynCom intervention. Subsequently, controlled pot experiments should be conducted to quantify hydraulic traits (embolism vulnerability, rehydration recovery rate) and nutrient indicators (rhizosphere available N/P increment, plant survival rate and biomass), thereby validating the synergistic benefits of the proposed framework. (3) The three core functional modules, rhizosphere remediation, root architecture remodeling, and stress tolerance regulation, can be proportionally tailored to the specific stress profiles of different land units, allowing for on-demand assembly and precisely targeted alleviation. Such customized microbial consortia are expected to mitigate the risk of community functional collapse under extreme environmental conditions, thereby facilitating the long-term sustainable restoration of desert vegetation.

## 7 Conclusion

Desert ecosystems are complex systems characterized by extreme water stress and intricate interactions across plant–microbe–soil interfaces. This review reveals that SynComs help seedling establishment through multiple synergies. We emphasize that the effectiveness of SynComs does not arise from the additive effects of individual strains, but from the initiation and maintenance of a plant-soil-microbe positive feedback loop. When this feedback operates stably, it progressively enhances SOC stocks, aggregate stability, and plant WUE; conversely, SynComs designs lacking ecological compatibility may lead to functional degradation or ecological invasion risks. Based on this, we propose a paradigm shift from strain combination to system design. First, integrate single-cell technologies and multi-omics approaches to mine the functional dark matter of uncultured desert microorganisms; on this foundation, employ GSMMs and ML to predict microbial interactions, thereby improving the stability and functionality of SynComs, and achieve iterative optimization through in situ ecological feedback validation, ultimately forming a closed-loop system of resource mining-rational design-in situ verification. In the future, these modular SynComs, by integrating multiple metabolic pathways, will provide more efficient solutions and technical support for vegetation restoration and reconstruction in desert ecosystems.

## CRediT authorship contribution statement

**Panpan Zhang:** Writing – review & editing, Writing – original draft, Visualization, Software, Conceptualization. **Haibing Wang:** Writing – review & editing, Supervision. **Xiaopeng Jia:** Writing – review & editing, Supervision. **Xiaopei Zhang:** Writing – review & editing.

## Data availability

No data was used for the research described in the article.

## Declaration of competing interest

The authors declare that they have no known competing financial interests or personal relationships that could have appeared to influence the work reported in this paper.

## References

[1] Eshel G., Araus V., Undurraga S. (2021). Plant ecological genomics at the limits of life in the atacama desert. Proc Natl Acad Sci USA.

[2] Islam W.Z., Khan Zhihao, Ali Khalid, Zeng Fanjiang (2026). Resilience and adaptation in desert ecosystems: unveiling microbial legacies and plant functional trait coordination under climate change. Glob Change Biol.

[3] Coleine C., Delgado-Baquerizo M., DiRuggiero J. (2024). Dryland microbiomes reveal community adaptations to desertification and climate change. ISME J.

[4] Leung P.M., Bay S.K., Meier D.V. (2020). Energetic basis of microbial growth and persistence in desert ecosystems. mSystems.

[5] Islam W., Naveed H., Zhihao Z. (2026). Desert ecosystems as carbon frontiers: innovations in sequestration and climate adaptation strategies. Biol Rev.

[6] Song J., Wan S., Zhang K. (2024). Ecological restoration enhances dryland carbon stock by reducing surface soil carbon loss due to wind erosion. Proc Natl Acad Sci USA.

[7] Islam W., Zeng F., Siddiqui J.A. (2025). Combating desertification: comprehensive strategies, challenges, and future directions for sustainable solutions. Biol Rev.

[8] Li C., Fu B., Wang S., Stringer L.C., Wang Y., Li Z., Liu Y., Zhou W. (2021). Drivers and impacts of changes in China’s drylands. Nat Rev Earth Environ.

[9] Islam W., Zeng F., Alotaibi M.O., Khan K.A. (2024). Unlocking the potential of soil microbes for sustainable desertification management. Earth Sci Rev.

[10] Ma Q., Liu H.-S., Li H.-J. (2024). Genomic analysis reveals phylogeny of Zygophyllales and mechanism for water retention of a succulent xerophyte. Plant Physiol.

[11] Han Y., Deng J., Zhou W. (2022). Seasonal responses of hydraulic function and carbon dynamics in spruce seedlings to continuous drought. Front Plant Sci.

[12] Torres-Ruiz J.M., Cochard H., Delzon S. (2024). Plant hydraulics at the heart of plant, crops and ecosystem functions in the face of climate change. New Phytol.

[13] Lu X., Zou M., Gan G. (2025). Excessive irrigation-driven greening has triggered water shortages and compromised sustainability. Agric Water Manag.

[14] Zhou H., Li W. (2015). Responses and adaptation of xylem hydraulic conductivity to salt stress in *Populus euphratica*. Chin J Plant Ecol.

[15] Araujo A.S.F., Pereira A.P.A., de Medeiros E.V. (2025). Soil microbes: below-ground defenders against desertification. Trends Ecol Evol.

[16] Dini-Andreote F., Wells D.M., Atkinson J.A. (2025). Microbial drivers of root plasticity. New Phytol.

[17] Xiang Q., Yang K., Cui L. (2025). Global exploration of drought-tolerant bacteria in the wheat rhizosphere reveals microbiota shifts and functional taxa enhancing plant resilience. Nat Food.

[18] Xie T., Lin Y.W., Li X.R. (2024). Responses of the arbuscular mycorrhizal fungi community to warming coupled with increased drought in an arid desert region. Geoderma.

[19] Guo P., Peng F., Xue X. (2025). Plants experience reduced a negative plant-soil feedback due to mycorrhizal fungi change with neighbors in a harsh environment. Biol Fertil Soils.

[20] Tang B., Man J., Romero F. (2024). Mycorrhization enhances plant growth and stabilizes biomass allocation under drought. Glob Change Biol.

[21] Alsharif W., Saad M.M., Hirt H. (2020). Desert microbes for boosting sustainable agriculture in extreme environments. Front Microbiol.

[22] Zhang J., Ma X., Gao Y. (2026). Salinity enhances soil organic carbon stability in hyper-arid deserts via increased mineral-association carbon and microbial carbon use efficiency. Land Degrad Dev.

[23] Zhang Y., Du Y., Zhang Z. (2024). Variation in root-associated microbial communities among three different plant species in natural desert ecosystem. Plants.

[24] Liu X., Chen Y., Lu J. (2025). Ultimate soil nitrogen microbial function evolution pathway fixation–comammox–nitrate reduction in long–term arid. Commun Earth Environ.

[25] Sun K., Yang R., Liu J. (2025). Precipitation changes reshape desert soil microbial community assembly and potential functions. Environ Res.

[26] Monger C., Sala O., Duniway M. (2015). Legacy effects in linked ecological–soil–geomorphic systems of drylands. Front Ecol Environ.

[27] Peng Z., Gao H., Pan H. (2024). Environmental change legacies attenuate disturbance response of desert soil microbiome and multifunctionality. Funct Ecol.

[28] Qi H., Wen X., Wang Z. (2026). Microbial memory of drought reshapes root-associated communities to enhance plant resilience. Plant Cell Environ.

[29] Angot V., Thiour-Mauprivez C., Schellenberger R. (2025). Alleviation of water-deficit inhibition of plant growth by rhizosphere microbiota conditioning. New Phytol.

[30] Ginnan N.A., Custódio V., Gopaulchan D. (2025). Precipitation legacy effects on soil microbiota facilitate adaptive drought responses in plants. Nat Microbiol.

[31] de-Bashan L.E., Hernandez J.-P., Bashan Y. (2012). The potential contribution of plant growth-promoting bacteria to reduce environmental degradation – A comprehensive evaluation. Appl Soil Ecol.

[32] Tang B., Man J., Lehmann A. (2024). Arbuscular mycorrhizal fungi attenuate negative impact of drought on soil functions. Glob Change Biol.

[33] Galaviz C., Lopez B.R., de-Bashan L.E. (2018). Root growth improvement of mesquite seedlings and bacterial rhizosphere and soil community changes are induced by inoculation with plant growth-promoting bacteria and promote restoration of eroded desert soil. Land Degrad Dev.

[34] Astorga-Eló M., Gonzalez S., Acuña J.J. (2021). Rhizobacteria from ‘flowering desert’ events contribute to the mitigation of water scarcity stress during tomato seedling germination and growth. Sci Rep.

[35] Hu D., Baskin J.M., Baskin C.C. (2019). Arbuscular mycorrhizal symbiosis and achene mucilage have independent functions in seedling growth of a desert shrub. J Plant Physiol.

[36] Al-Yahya’ei M.N., Błaszkowski J., Al-Hashmi H. (2022). From isolation to application: a case study of arbuscular mycorrhizal fungi of the Arabian Peninsula. Symbiosis.

[37] Pan J., Huang C., Peng F. (2020). Effect of Arbuscular Mycorrhizal Fungi (AMF) and plant growth-promoting bacteria (PGPR) inoculations on *Elaeagnus angustifolia* L. in saline soil. Appl Sci.

[38] Li C., Han Y., Zou X. (2024). A systematic discussion and comparison of the construction methods of synthetic microbial community. Synth Syst Biotechnol.

[39] Mehlferber E.C., Arnault G., Joshi B. (2024). A cross-systems primer for synthetic microbial communities. Nat Microbiol.

[40] Zhang Y., Jing M., Lyu L. (2026). Principles for rigorous design and application of synthetic microbial communities. Adv Sci.

[41] Xu X., Dinesen C., Pioppi A. (2025). Composing a microbial symphony: synthetic communities for promoting plant growth. Trends Microbiol.

[42] Qiao Y., Wang Z., Sun H. (2024). Synthetic community derived from grafted watermelon rhizosphere provides protection for ungrafted watermelon against *Fusarium oxysporum* via microbial synergistic effects. Microbiome.

[43] Li Z., Bai X., Jiao S. (2021). A simplified synthetic community rescues Astragalus mongholicus from root rot disease by activating plant-induced systemic resistance. Microbiome.

[44] Liu C., Jiang M., Yuan M.M. (2023). Root microbiota confers rice resistance to aluminium toxicity and phosphorus deficiency in acidic soils. Nat Food.

[45] Zhang L., Zhang M., Huang S. (2022). A highly conserved core bacterial microbiota with nitrogen-fixation capacity inhabits the xylem sap in maize plants. Nat Commun.

[46] Zhou X., Wang J., Liu F. (2022). Cross-kingdom synthetic microbiota supports tomato suppression of Fusarium wilt disease. Nat Commun.

[47] Niu B., Paulson J.N., Zheng X., Kolter R. (2017). Simplified and representative bacterial community of maize roots. Proc Natl Acad Sci U S A.

[48] Schmitz L., Yan Z., Schneijderberg M. (2022). Synthetic bacterial community derived from a desert rhizosphere confers salt stress resilience to tomato in the presence of a soil microbiome. ISME J.

[49] Hao X., Wang X., Wang X. (2026). Synthetic community derived from the root core microbes of a desert shrub *Caragana korshinskii* enhances wheat drought tolerance. Microbiome.

[50] Hosseyni Moghaddam M.S., Safaie N., Soltani J. (2021). Desert-adapted fungal endophytes induce salinity and drought stress resistance in model crops. Plant Physiol Biochem.

[51] Tariq A., Gao Y., Zeng F. (2026). Guardians of arid lands: deep-rooted defense against desertification and climate change. Trends Plant Sci.

[52] Cavanaugh M.L., Kurc S.A., Scott R.L. (2011). Evapotranspiration partitioning in semiarid shrubland ecosystems: a two-site evaluation of soil moisture control on transpiration. Ecohydrology.

[53] Niu F., Chen J., Xiong P. (2019). Responses of soil respiration to rainfall pulses in a natural grassland community on the semi-arid Loess Plateau of China. Catena.

[54] Song W., Chen S., Zhou Y. (2020). Rainfall amount and timing jointly regulate the responses of soil nitrogen transformation processes to rainfall increase in an arid desert ecosystem. Geoderma.

[55] Tariq A., Graciano C., Sardans J. (2024). Plant root mechanisms and their effects on carbon and nutrient accumulation in desert ecosystems under changes in land use and climate. New Phytol.

[56] He L., Guo J., Xiong W. (2023). Sand-fixing vegetation regulates deep percolation and soil water dynamics in semiarid sandy land: evidence from a deep percolation recorder and in-situ test. Catena.

[57] Hao X.-m., Li C., Guo B. (2012). Dew formation and its long-term trend in a desert riparian forest ecosystem on the eastern edge of the taklimakan desert in China. J Hydrol.

[58] Kidron G.J. (1999). Altitude dependent dew and fog in the Negev Desert, Israel. Agric For Meteorol.

[59] Chen X., Wu Y., Hai C. (2023). Effects of surface dew under different types of sand-fixing shrubs in the southern margin of Mu us sandy land, Northern China. J Desert Res.

[60] Pan Y., Wang X. (2014). Effects of shrub species and microhabitats on dew formation in a revegetation-stabilized desert ecosystem in Shapotou, northern China. J Arid Land.

[61] Ran B., Zhang Z., Yang J. (2023). Dew formation characteristics of *Artemisia ordosica* and its influence on water balance in the Mu us Sandy Land. Trans Chin Soc Agric Eng.

[62] Querejeta J., Prieto I., Armas C. (2022). Higher leaf nitrogen content is linked to tighter stomatal regulation of transpiration and more efficient water use across dryland trees. New Phytol.

[63] Wang S., Ciais P., Reich P.B. (2025). Phosphorus constrains global photosynthesis more than nitrogen does. Nat Ecol Evol.

[64] Querejeta J.I., Ren W., Prieto I. (2021). Vertical decoupling of soil nutrients and water under climate warming reduces plant cumulative nutrient uptake, water-use efficiency and productivity. New Phytol.

[65] Gessler A., Schaub M., McDowell N.G. (2017). The role of nutrients in drought-induced tree mortality and recovery. New Phytol.

[66] Zhang Z., Yano K. (2026). Miscanthus sinensis dominates in andosol through strong phosphorus acquisition that enhances water-use efficiency and nitrogen fixation. Front Plant Sci.

[67] He P., Sardans J., Wang X. (2024). Nutritional changes in trees during drought-induced mortality: a comprehensive meta-analysis and a field study. Glob Change Biol.

[68] Šimanský V., Juriga M., Jonczak J. (2019). How relationships between soil organic matter parameters and soil structure characteristics are affected by the long-term fertilization of a sandy soil. Geoderma.

[69] Ankenbauer K.J., Loheide II S.P. (2017). The effects of soil organic matter on soil water retention and plant water use in a meadow of the Sierra Nevada, CA. Hydrol Process.

[70] Fu Z., Hu W., Beare M.H. (2021). Contributions of soil organic carbon to soil water repellency persistence: characterization and modelling. Geoderma.

[71] Hakobyan A., Velte S., Sickel W. (2023). Tillandsia landbeckii phyllosphere and laimosphere as refugia for bacterial life in a hyperarid desert environment. Microbiome.

[72] Dong L., Li M.-X., Li S. (2024). Aridity drives the variability of desert soil microbiomes across north-western China. Sci Total Environ.

[73] Duan Y., Wang X., Wang L. (2022). Biogeographic patterns of soil microbe communities in the deserts of the hexi corridor, northern China. Catena.

[74] Delgado-Baquerizo M., Oliverio A.M., Brewer T.E. (2018). A global atlas of the dominant bacteria found in soil. Science.

[75] Li M.-X., Lian W.-H., Lian Z.-H. (2025). Rare and abundant taxa in *Artemisia desertorum* rhizosphere soils demonstrate disparate responses to drought stress. Adv Biotechnol.

[76] Du Y., Zhang L., Yang Y. (2025). Assembly, network and functional compensation of specialists and generalists in poplar rhizosphere under salt stress. npj Biofilms Microbiomes.

[77] Mousa W.K., AlShami R., Ghemrawi R. (2026). Plant microbiome of the arabian peninsula desert reveals unique structural and functional adaptations supporting climate resilience. F1000Research.

[78] Yang Y., Deng Y., Cai C. (2025). Biocrusts' impact on hydrological processes and erosion dynamics: a review. Geoderma.

[79] Li T., Fang Y., Fan Y. (2025). Migration and structural implication of Cyanobacteria in biological soil crusts in response to water and particle burial. Innovation.

[80] Coban O., De Deyn G.B., van der Ploeg M. (2022). Soil microbiota as game-changers in restoration of degraded lands. Science.

[81] Li Y.-G., Huang Y.-J., Zhang Q. (2025). Surface soil physical properties and stability: determined by biological soil crust type and driven by aridity in the desert regions of Northwest China. Soil Sci Soc Am J.

[82] Cordero I., Leizeaga A., Hicks L.C. (2023). High intensity perturbations induce an abrupt shift in soil microbial state. ISME J.

[83] Pantigoso H.A., Ossowicki A., Stringlis I.A. (2025). Hub metabolites at the root–microbiome interface: unlocking plant drought resilience. Trends Plant Sci.

[84] Maphosa S., Steyn M., Lebre P.H. (2025). Rhizosphere bacterial communities of namib desert plant species: evidence of specialised plant-microbe associations. Microbiol Res.

[85] Barnard R.L., Osborne C.A., Firestone M.K. (2013). Responses of soil bacterial and fungal communities to extreme desiccation and rewetting. ISME J.

[86] Makhalanyane T.P., Valverde A., Gunnigle E. (2015). Microbial ecology of hot desert edaphic systems. FEMS (Fed Eur Microbiol Soc) Microbiol Rev.

[87] Schimel J.P. (2018). Life in dry soils: effects of drought on soil microbial communities and processes. Annu Rev Ecol Evol Syst.

[88] Metze D., Schnecker J., Canarini A. (2023). Microbial growth under drought is confined to distinct taxa and modified by potential future climate conditions. Nat Commun.

[89] Ren Y., Gao G.-L., Ding G.-D. (2023). Species composition and driving factors of the ectomycorrhizal fungal community associated with *Pinus sylvestris* var. *mongolica* at different growth periods. Chin J Plant Ecol.

[90] Jia Y., Qin W., Zhang T. (2023). Progress on mechanisms underlying arbuscular mycorrhizal fungi maintaining desert ecosystem stability under climate change. Chin Sci Bull.

[91] Shi Z., Gao S., Wang F. (2008). Biodiversity of arbuscular mycorrhizal fungi in desert ecosystems. Arid Zone Res.

[92] Zhou S., Guo L. (2021).

[93] Porto D., Arauco A., Boechat C. (2020). Arbuscular mycorrhizal fungi on the inital growth and nutrition of *Parkia Platycephala* benth. under water stress. Cerne.

[94] Souza R.G., Silva D.K.A., Oliveira J.R.G. (2012). Use of mycorrhizal seedlings on recovery of mined dunes in northeastern Brazil. Pedobiologia.

[95] Wu C., Bi Y., Peng S. (2026). Overcoming water limitation in Hippophae rhamnoides: AMF symbiosis optimizes root development and water use efficiency under arid conditions. Rhizosphere.

[96] Chen Z., Shi Z., Tian C. (2008). Effects of arbuscular mycorrhizal fungal inoculation on growth and nutrient uptake of two ephemeral plants. Chin J Plant Ecol.

[97] Akachoud O., Raho O., Hazzoumi Z., Idbella M. (2026). The soil microbiome in agriculture: opportunities and challenges.

[98] Qian W., He X. (2009). Diversity of arbuscular mycorrhizal fungi associated with a desert plant Artemisia ordosica. Biodivers Sci.

[99] Fu W., Yu Q., Zhang H. (2026). Beyond plant diversity: plant productivity responses to extreme drought are linked to arbuscular Mycorrhizal Fungal diversity. Glob Change Biol.

[100] Qiu D., Xiao B., Saez-Sandino T. (2026). Biocrusts are highly vulnerable to multidimensional global change. Glob Change Biol.

[101] Belsey S., Shoseyov O., Zer H. (2025). A hybrid cellulose nano-crystals cyanobacterial living material improves soil crust rehabilitation. Environ Technol Innov.

[102] Zhang S., Haiyan G., Deren Yan, Huang Haiguang (2023). Effects of desert biological soil crusts succession on microbial community structure and soil enzyme activities. J Desert Res.

[103] Sun X., Xu Z., Zhang N. (2026). Bacillus velezensis SQR9: a model biofertilizer strain for beneficial plant root-rhizobacterium interaction. Sci China Life Sci.

[104] de Vries F.T., Griffiths R.I., Knight C.G. (2020). Harnessing rhizosphere microbiomes for drought-resilient crop production. Science.

[105] Chen C., Zhan J., Du W. (2025). The mechanisms of plant-associated microbes in regulating plant drought adaptation. J Plant Ecol.

[106] Chen Y., Neilson J.W., Kushwaha P. (2021). Life-history strategies of soil microbial communities in an arid ecosystem. ISME J.

[107] Ray A.E., Zaugg J., Benaud N. (2022). Atmospheric chemosynthesis is phylogenetically and geographically widespread and contributes significantly to carbon fixation throughout cold deserts. ISME J.

[108] Jordaan K., Lappan R., Dong X. (2020). Hydrogen-oxidizing bacteria are abundant in desert soils and strongly stimulated by hydration. mSystems.

[109] Andreani-Gerard C.M., Jiménez N.E., Palma R., Muller C., Hamon-Giraud P., Le Cunff Y., Cambiazo V., González M., Siegel A., Frioux C., Maass A. (2025). Modeling the emergent metabolic potential of soil microbiomes in Atacama landscapes. Environ Microbiome.

[110] Philippot L., Chenu C., Kappler A. (2024). The interplay between microbial communities and soil properties. Nat Rev Microbiol.

[111] Kakouridis A., Hagen J.A., Kan M.P. (2022). Routes to roots: direct evidence of water transport by arbuscular mycorrhizal fungi to host plants. New Phytol.

[112] Griffin C., Oz M.T., Demirer G.S. (2024). Engineering plant–microbe communication for plant nutrient use efficiency. Curr Opin Biotechnol.

[113] Trivedi P., Leach J.E., Tringe S.G. (2020). Plant–microbiome interactions: from community assembly to plant health. Nat Rev Microbiol.

[114] El-Keblawy A., Jarrar H., Manikandan S.K. (2025). Rhizosheaths in desert plants: natural mechanisms and bioinspired technologies for water harvesting and dryland restoration. Sci Total Environ.

[115] Wang B., An S., Liang C. (2021). Microbial necromass as the source of soil organic carbon in global ecosystems. Soil Biol Biochem.

[116] He A., Niu S., Yang D. (2021). Two PGPR strains from the rhizosphere of haloxylon ammodendron promoted growth and enhanced drought tolerance of ryegrass. Plant Physiol Biochem.

[117] Ye Q., Wang H., Li H. (2023). Arbuscular mycorrhizal fungi enhance drought stress tolerance by regulating osmotic balance, the antioxidant system, and the expression of drought-responsive genes in *Vitis vinifera* L. Aust J Grape Wine Res.

[118] Liu F., Ma H., Liu B. (2023). Effects of plant growth-promoting rhizobacteria on the physioecological characteristics and growth of walnut seedlings under drought stress. Agronomy.

[119] Francioli D., Kampouris I.D., Kuhl-Nagel T. (2025). Microbial inoculants modulate the rhizosphere microbiome, alleviate plant stress responses, and enhance maize growth at field scale. Genome Biol.

[120] Li L., Cheng K., Du Y. (2025). Rhizosphere microbes from *Populus euphratica* conferred salt stress resistance to *Populus alba* × *Populus glandulosa*. Plant Cell Environ.

[121] Liu X., Wang B., Jiang J. (2025). Drought-tolerant rhizobacteria boost crop resilience. Nat Food.

[122] Li J., Liu H., Wang J. (2025). Drought-induced plant microbiome and metabolic enrichments improve drought resistance. Cell Host Microbe.

[123] Niu G., Liu W., Zhang T. (2025). Deciphering conserved rhizosphere metabolite–microbiome interactions for crop drought resistance. Glob Change Biol.

[124] Affortit P., Ahmed M.A., Grondin A. (2023). Keep in touch: the soil–root hydraulic continuum and its role in drought resistance in crops. J Exp Bot.

[125] Chávez-González J.D., Flores-Núñez V.M., Merino-Espinoza I.U. (2024). Desert plants, arbuscular mycorrhizal fungi and associated bacteria: exploring the diversity and role of symbiosis under drought. Environ Microbiol Rep.

[126] Shi J., Wang X., Wang E. (2023). Mycorrhizal symbiosis in plant growth and stress adaptation: from genes to ecosystems. Annu Rev Plant Biol.

[127] Johns N.I., Blazejewski T., Gomes A.L.C. (2016). Principles for designing synthetic microbial communities. Curr Opin Microbiol.

[128] Großkopf T., Soyer O.S. (2014). Synthetic microbial communities. Curr Opin Microbiol.

[129] Lawson C.E., Harcombe W.R., Hatzenpichler R. (2019). Common principles and best practices for engineering microbiomes. Nat Rev Microbiol.

[130] Ruan Z., Chen K., Cao W. (2024). Engineering natural microbiomes toward enhanced bioremediation by microbiome modeling. Nat Commun.

[131] Li S., Lian W.-H., Han J.-R. (2023). Capturing the microbial dark matter in desert soils using culturomics-based metagenomics and high-resolution analysis. npj Biofilms Microbiomes.

[132] Yang Z.-W., Lian Z.-H., Liu L. (2023). Cultivation strategies for prokaryotes from extreme environments. iMeta.

[133] Wang Z., Wang S., He Q. (2025). Ecological design of high-performance synthetic microbial communities: from theoretical foundations to functional optimization. ISME Commun.

[134] Diao Z., Peng Q., Luo S. (2025). AI-powered high-throughput digital colony picker platform for sorting microbial strains by multi-modal phenotypes. Nat Commun.

[135] Hitch T.C.A., Bosch J., Bolsega S. (2025). Function-based selection of synthetic communities enables mechanistic microbiome studies. bioRxiv.

[136] Miller T., Mikiciuk G., Kisiel A. (2023). Machine learning approaches for forecasting the best microbial strains to alleviate drought impact in agriculture. Agriculture.

[137] Zhou T., Zhao F. (2025). AI-empowered human microbiome research. Gut.

[138] Jing J., Garbeva P., Raaijmakers J.M. (2024). Strategies for tailoring functional microbial synthetic communities. ISME J.

[139] De la Vega-Camarillo E., Arreola-Vargas J., Antony-Babu S. (2025). Machine learning-guided synthetic microbial communities enable functional and sustainable degradation of persistent environmental pollutants. bioRxiv.

[140] Tawfiq R., Niu K., Hoehndorf R. (2024). DeepGOMeta for functional insights into microbial communities using deep learning-based protein function prediction. Sci Rep.

[141] Wang X., Li M., Wang W., Wang S., Chen S. (2021). Effect of single cell and Filamentous Microorganisms on Formation of Soil Aggregates. Chin J Soil Sci.

[142] Ji L., Chen X., Huang C. (2024). Arbuscular mycorrhizal hyphal networks and glomalin-related soil protein jointly promote soil aggregation and alter aggregate hierarchy in Calcaric Regosol. Geoderma.

[143] Liu W., Liu R., Tang Z. (2026). Dual-beneficial synergistic Trichoderma-bacterial cross-kingdom SynCom facilitates *Medicago sativa* productivity and soil organic carbon sequestration in saline-alkaline soil. Plant Soil.

[144] Zhou X., Liang B., Zhang T. (2024). Co-inoculation of fungi and desert Cyanobacteria facilitates biological soil crust formation and soil fertility. Front Microbiol.

[145] Li Q., Zhu P., Tian G. (2026). Synthetic microbial communities enhance artificial cyanobacterial crusts formation via spatiotemporal synergy. Microorganisms.

[146] Gonin M., Salas-González I., Gopaulchan D. (2023). Plant microbiota controls an al-ternative root branching regulatory mechanism in plants. Proc Natl Acad Sci U S A.

[147] Finkel O.M., Salas-González I., Castrillo G. (2020). A single bacterial genus maintains root growth in a complex microbiome. Nature.

[148] Armanhi J.S.L., de Souza R.S.C., Biazotti B.B. (2021). Modulating drought stress response of maize by a synthetic bacterial community. Front Microbiol.

[149] Zhang C., van der Heijden M.G.A., Dodds B.K. (2024). A tripartite bacterial-fungal-plant symbiosis in the mycorrhiza-shaped microbiome drives plant growth and mycorrhization. Microbiome.

[150] Khan W., Zhu Y., Khan A. (2024). Above-and below-ground feedback loop of maize is jointly enhanced by plant growth-promoting rhizobacteria and arbuscular mycorrhizal fungi in drier soil. Sci Total Environ.

[151] Li S., Li P., Ding H. (2025). Rhizosphere bacterial community confers drought tolerance to *Astragalus mongholicus*. Agric Water Manag.

[152] Gao H., Huang Z., Chen W. (2024). Mild to moderate drought stress reinforces the role of functional microbiome in promoting growth of a dominant forage species (*Neopallasia pectinata*) in desert steppe. Front Microbiol.

[153] Liu S., Wu J., Cheng Z. (2025). Microbe-mediated stress resistance in plants: the roles played by core and stress-specific microbiota. Microbiome.

[154] Zi H., Hua Z., Wang Y. (2025). Mycorrhizal colonization of dryland tree establishment depends on soil microbial cooperation. Nat Commun.

[155] Hao X., Wang X., Chen C. (2023). Synthetic bacterial communities reshape microbial communities and enhance nutrient supply in desertified land of Northwest China. Appl Soil Ecol.

[156] Hao X., Gu Y., Zhang H. (2024). Synthetic microbial community promotes bacterial communities leading to soil multifunctionality in desertified land. Microorganisms.

[157] Asif K., Shabaan M., Mahmood W. (2024). Synergistic application of bacterial consortium and organic amendments improves the growth and seed quality of mash bean (*Vigna Mungo* L.). J Soil Sci Plant Nutr.

[158] de Goede S.P.C., Hannula S.E., Jansen B. (2025). Fungal-mediated soil aggregation as a mechanism for carbon stabilization. ISME J.

[159] Chamizo S., Mugnai G., Rossi F. (2018). Cyanobacteria inoculation improves soil stability and fertility on different textured soils: gaining insights for applicability in soil restoration. Front Environ Sci.

[160] Ali S.K., Hozzein W.N., Korany S.M., Ahmed M.E., Hamed E.A., Mohamed H.S. (2025). Improvement of water retention capacity in sandy soil with the application of a crude exopolysaccharide-containing fermentation broth produced by *Kocuria carniphila* AUMC B-453. World J Microbiol Biotechnol.

[161] Flemming H.-C., Wingender J. (2010). The biofilm matrix. Nat Rev Microbiol.

[162] Baveye P.C. (2020). “Soil biofilms”: misleading description of the spatial distribution of microbial biomass in soils. Soil Ecol Lett.

[163] Alghanmi L., Elhady A., Parween S. (2025). Plant growth promotion and pathogen protection by the desert endophyte *Pseudomonas grandensis* R4-79. BMC Microbiol.

[164] Yang N., Nesme J., Røder H.L. (2021). Emergent bacterial community properties induce enhanced drought tolerance in *A**rabidopsis*. npj Biofilms Microbiomes.

[165] Yang N., Røder H.L., Wicaksono W.A. (2024). Interspecific interactions facilitate keystone species in a multispecies biofilm that promotes plant growth. ISME J.

[166] Morris E.K., Morris D.J.P., Vogt S. (2019). Visualizing the dynamics of soil aggregation as affected by arbuscular mycorrhizal fungi. ISME J.

[167] Don A., Seidel F., Leifeld J. (2024). Carbon sequestration in soils and climate change mitigation—Definitions and pitfalls. Glob Change Biol.

[168] Kang N.K., Koh H.G., Choi Y. (2025). Bioconversion of CO2 into valuable bioproducts via synthetic modular co-culture of engineered *Chlamydomonas reinhardtii* and *Escherichia coli*. Metab Eng.

[169] Shi K., Zheng Q., Wang B. (2026). Continental-scale drivers of soil microbial extracellular polymeric substances. Nat Commun.

[170] Chowaniec K., Zubek S., Skubała K. (2025). Exopolysaccharides in biological soil crusts are important contributors to carbon and nutrient storage after the restoration of inland sand dunes. Plant Soil.

[171] Rawls W.J., Pachepsky Y.A., Ritchie J.C. (2003). Effect of soil organic carbon on soil water retention. Geoderma.

[172] Xie Y., Wen X., Tu Y. (2024). Mechanisms of artificial biological soil crusts development for anti-desertification engineering on the Qinghai-Tibetan Plateau. Environ Technol Innov.

[173] Zhang J., Zhao R., Li X. (2024). Potential of arbuscular mycorrhizal fungi for soil health: a review. Pedosphere.

[174] Liu H., Li J., Singh B.K. (2024). Harnessing co-evolutionary interactions between plants and streptomyces to combat drought stress. Nat Plants.

[175] Liu Y., Shi A., Chen Y. (2025). Beneficial microorganisms: regulating growth and defense for plant welfare. Plant Biotechnol J.

[176] Zhang J., Feng Y., Maestre F.T. (2023). Water availability creates global thresholds in multidimensional soil biodiversity and functions. Nat Ecol Evol.

[177] Qi J., Liang C., Zhang C. (2026). Intensifying aridity undermines the role of soil biodiversity in supporting ecosystem stability. Glob Change Biol.

[178] Zhou T., Delgado-Baquerizo M., Ren C. (2025). Soil microbial life history strategies co-vary with ecosystem multifunctionality across aridity gradients. Proc Natl Acad Sci U S A.

[179] Najafi Zilaie M., Mosleh Arani A., Etesami H. (2022). Halotolerant rhizobacteria enhance the tolerance of the desert halophyte Nitraria schoberi to salinity and dust pollution by improving its physiological and nutritional status. Appl Soil Ecol.

[180] Xie L., Yang Y., Ma J. (2024). Variations in ectomycorrhizal exploration types parallel seedling fine root traits of two temperate tree species under extreme drought and contrasting solar radiation treatments. Plant Cell Environ.

[181] Cosme M. (2023). Mycorrhizas drive the evolution of plant adaptation to drought. Commun Biol.

[182] Shi Z., Mickan B., Feng G. (2015). Arbuscular mycorrhizal fungi improved plant growth and nutrient acquisition of desert ephemeral Plantago minuta under variable soil water conditions. J Arid Land.

[183] Zhang T., Sun Y., Song Y. (2011). On-site growth response of a desert ephemeral plant, Plantago minuta, to indigenous arbuscular mycorrhizal fungi in a central Asia desert. Symbiosis.

[184] Duan S., Jin Z., Zhang L. (2025). Mechanisms of cooperation in the plants-arbuscular mycorrhizal fungi-bacteria continuum. ISME J.

[185] Mažylytė R., Kailiuvienė J., Mažonienė E. (2024). The Co-Inoculation effect on Triticum aestivum growth with synthetic microbial communities (SynComs) and their potential in agrobiotechnology. Plants.

[186] Wang J., Li R., Zhang H. (2020). Beneficial bacteria activate nutrients and promote wheat growth under conditions of reduced fertilizer application. BMC Microbiol.

[187] Emami-Karvani Z., Chitsaz-Esfahani Z., Yadav A.N. (2021). Soil microbiomes for sustainable agriculture: functional annotation.

[188] Xiao K.-Q., Ge T.-D., Wu X.-H. (2021). Metagenomic and 14C tracing evidence for autotrophic microbial CO2 fixation in paddy soils. Environ Microbiol.

[189] Xie T., Jia P., Lin Y. (2025). Warming modulates soil multifunctionality through assembly processes and co-occurrence patterns of arbuscular mycorrhizal fungal communities in drylands. Funct Ecol.

[190] Rehman M.M.U., Xiao Y.-L., Khattak S. (2026). The interactions between rhizobacteria and arbuscular mycorrhizal fungi enhance global soil carbon sequestration in drylands: a global meta-analysis. J Adv Res.

[191] Duan S., Feng G., Limpens E. (2024). Cross-kingdom nutrient exchange in the plant–arbuscular mycorrhizal fungus–bacterium continuum. Nat Rev Microbiol.

[192] Zhang L., Zhou J., George T.S. (2022). Arbuscular mycorrhizal fungi conducting the hyphosphere bacterial orchestra. Trends Plant Sci.

[193] Coker J., Zhalnina K., Marotz C. (2022). A reproducible and tunable synthetic soil microbial community provides new insights into microbial ecology. mSystems.

[194] Wang Y., Wang Z., Liu Y. (2025). Assembly processes, ecological strategies, and functional potentials of abundant and rare microbial taxa in biological soil crusts from two habitats in arid ecosystems. Eur J Soil Biol.

[195] Hou Q., Xia R., Yuan B. (2025). Divergent community assembly processes and multifunctionality contributions of abundant and rare soil bacteria during a 53-year restoration in the tengger desert, China. Commun Biol.

[196] Delgado-Baquerizo M. (2022). Simplifying the complexity of the soil microbiome to guide the development of next-generation SynComs. J Sustain Agric Environ.

[197] Bull A., Asenjo J., Goodfellow M. (2016). The Atacama desert: technical resources and the growing importance of novel microbial diversity. Annu Rev Microbiol.

[198] Jiao J.-Y., Liu L., Hua Z.-S. (2020). Microbial dark matter coming to light: challenges and opportunities. Natl Sci Rev.

[199] Bang C., Dagan T., Deines P. (2018). Metaorganisms in extreme environments: do microbes play a role in organismal adaptation?. Zoology.

[200] Lewis W.H., Tahon G., Geesink P. (2021). Innovations to culturing the uncultured microbial majority. Nat Rev Microbiol.

[201] Yu L., Luo X., Liu M. (2013). Diversity of ionizing radiation-resistant bacteria obtained from the taklimakan desert. J Basic Microbiol.

[202] van den Berg N.I., Machado D., Santos S. (2022). Ecological modelling approaches for predicting emergent properties in microbial communities. Nat Ecol Evol.

[203] Lin Y., Lin H., Liu J. (2024). Insights into constructing a stable and efficient microbial consortium system. Chin J Chem Eng.

[204] Gao Y., Peng K., Bai D. (2024). The Microbiome Protocols eBook initiative: building a bridge to microbiome research. iMeta.

[205] Liu L., Ding D., Wang H. (2025). Balancing cell growth and product synthesis for efficient microbial cell factories. Adv Sci.

[206] Silverstein M.R., Segrè D., Bhatnagar J.M. (2023). Environmental microbiome engineering for the mitigation of climate change. Glob Change Biol.

[207] Han J., Li S., Li W. (2024). Mining microbial and metabolic dark matter in extreme environments: a roadmap for harnessing the power of multi-omics data. Adv Biotech.

[208] de Souza R.S.C., Armanhi J.S.L., Arruda P. (2020). From microbiome to traits: designing synthetic microbial communities for improved crop resiliency. Front Plant Sci.

[209] Copeland C., Schulze-Lefert P., Ma K.-W. (2025). Potential and challenges for application of microbiomes in agriculture. Plant Cell.

[210] Wang G., van der Putten W.H., Klironomos J. (2025). Steering plant-soil feedback for sustainable agriculture. Science.

[211] Feng Q., Luo Y., Liang M. (2025). Rhizobacteria protective hydrogel to promote plant growth and adaption to acidic soil. Nat Commun.

[212] Su C., Jiang S., Dai J. (2025). Development of a versatile synthetic microbe for the concurrent degradation of organic pollutants. Adv Biotech.

[213] Martins S., Pasche J., Silva H. (2023). The use of synthetic microbial communities (SynComs) to improve plant health. Phytopathology.

[214] Rottinghaus A.G., Vo S., Moon T.S. (2023). Computational design of CRISPR guide RNAs to enable strain-specific control of microbial consortia. Proc Natl Acad Sci U S A.

[215] Folorunso T.R., Akwabeng P.M., Rowaiye A.B. (2026). High-throughput design of defined microbial consortia for crop protection. Ann Bot.

[216] Jorrin B., Haskett T.L., Knights H.E. (2024). Stable, fluorescent markers for tracking synthetic communities and assembly dynamics. Microbiome.

[217] Velte J.M., Mudiyanselage S., Hofmann O.F. (2025). Interactions between native soil microbiome and a synthetic microbial community reveals bacteria with persistent traits. mSystems.

